# A systematic comparison of transformers and ConvNets for root segmentation across nine datasets

**DOI:** 10.1186/s13007-026-01533-6

**Published:** 2026-04-20

**Authors:** Abraham George Smith, Sotiris Lamprinidis, Anand Seethepalli, Larry M. York, Eusun Han, Patrick Möhl, Kyriaki Boulata, Kristian Thorup-Kristensen, Jens Petersen

**Affiliations:** 1 Department of Computer Science, University of Copenhagen, Copenhagen, Denmark; 2 Department of Agroecology – Climate and Water, Aarhus University, Aarhus, Denmark; 3 Lancaster Environment Centre, Lancaster University, Lancaster, UK; 4 Department of Plant and Environmental Sciences, University of Copenhagen, Copenhagen, Denmark; 5 Oak Ridge National Laboratory, Oak Ridge, TN USA

**Keywords:** Root segmentation, Deep learning, Transformer, Convolutional neural network, Plant phenotyping, Image analysis, Transfer learning, Benchmark, Minirhizotron

## Abstract

**Background:**

Root segmentation is a fundamental yet challenging task in image-based plant phenotyping. Accurate segmentation is a prerequisite for extracting root traits relevant to plant physiology, breeding, and agronomy. While U-Net and other convolutional neural network (ConvNet) architectures have been applied to root segmentation, no systematic comparison of multiple Transformer and ConvNet architectures has been conducted across diverse root imaging conditions.

**Results:**

We evaluated 21 segmentation architectures across nine diverse root image datasets, training 1511 models to assess all combinations of architecture, dataset, pre-training strategy, and learning rate, producing over 3 million segmentations for evaluation. Transformer-based models significantly outperformed ConvNets for Dice (mean Dice 0.679 vs 0.659; $p = 3.0 \times 10^{-3}$). Root-diameter and root-length correlation were also higher for Transformers, but the differences were not statistically significant ($p = 0.054$ and $p = 0.198$ respectively). Pre-training significantly improved mean Dice from 0.623 to 0.666 ($p = 6.6 \times 10^{-10}$), with Transformers benefiting more from pre-training than ConvNets (Dice improvement + 0.072 vs + 0.021; $p = 3.7 \times 10^{-4}$), supporting the hypothesis that fine-tuned Transformers transfer more effectively across large domain gaps. MobileSAM achieved the highest Dice score (0.693) while maintaining computational efficiency. Both architecture families underestimated thin root length compared to manual annotations. Dataset choice explained 70.9% of performance variance, far exceeding model architecture (6.7%).

**Purpose:**

Transformer architectures significantly outperform ConvNets for root segmentation accuracy, and pre-training significantly improves performance, particularly for Transformers. Pre-trained MobileSAM offers the best accuracy at competitive computational cost. Dataset choice dominates performance variance, suggesting practitioners should prioritize data curation over architecture selection.

**Supplementary Information:**

The online version contains supplementary material available at 10.1186/s13007-026-01533-6.

## Background

Image-based methods are increasingly used to quantify plant root traits [1]. Segmentation of images into roots and background is a prerequisite for the extraction of root traits relevant to plant physiology [2], breeding [3], and agronomy [4].

Smith et al. [5] demonstrated U-Net’s [6] efficacy for the segmentation of chicory root images in soil. Their solution used images from a controlled Rhizobox setup [7].

More recently, complex root datasets presenting variation and artifacts not yet solved by U-Net [8] have highlighted that fully automatic root segmentation remains challenging. Even for less challenging Rhizotron datasets, treatment effects or genotype differences may be subtle and only revealed by sufficiently accurate segmentation models. Baykalov et al. [9] compared segmentation methods across diverse rhizotron datasets, finding that U-Net architectures achieved the best accuracy but that heterogeneous datasets remained challenging.

Transformer-based architectures have recently been applied to root segmentation. Li et al. [10] showed that Swin-Unet++ outperformed U-Net by 1.08% mIoU on cabbage seedling images, and Zhou et al. [11] applied Point Transformers to 3D Arabidopsis root point clouds. For ConvNet comparisons, Shen et al. [12] found that an improved DeepLabv3+ outperformed U-Net for cotton root segmentation from Minirhizotron images. However, these studies each evaluated one or two architectures on a single dataset. No systematic comparison of multiple Transformer and ConvNet architectures has been conducted across diverse root imaging conditions.

We hypothesise that: (H1) Transformer architectures outperform ConvNets for root segmentation, and (H2) pre-training improves performance for both architecture families compared to training from scratch.

To test these hypotheses, we evaluate 21 architectures across nine root image datasets, comparing pre-trained models to models trained from scratch. We include vision foundation models (MobileSAM, SAM2) and recent ConvNets (U-Net++, DeepLabV3+, MAnet). In total, we trained 1,511 models to assess all combinations of architecture, dataset, pre-training, and learning rate, producing over 3 million segmentations for evaluation.

## Results

Table 1 addresses H1 (Transformer vs ConvNet), Table 2 addresses H2 (pre-training), and Table 3 ranks all 21 models individually.

### Transformers outperform ConvNets

**Fig. 1:**
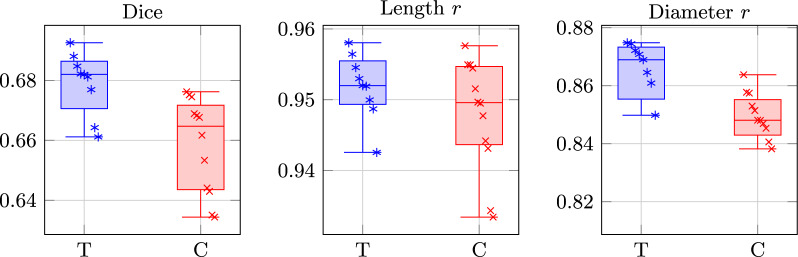
Per-model test performance by architecture family. T = Transformer, C = ConvNet. Left: Dice. Middle: Length *r*. Right: Diameter *r*. *r* = Pearson correlation coefficient. (One value per model; configuration selected by mean validation Dice across datasets)

Transformer models had significantly higher mean test Dice than ConvNets (0.679 vs 0.659; $p = 3.0 \times 10^{-3}$; Table 1). This difference is visible in Fig. 1 (left). Training and validation curves for all models are provided in Additional file 9. MobileSAM ViT-T, M2F Swin-S, and M2F Swin-T had the highest individual Dice values, occupying the top three positions in the ranking (Table 3). Several ConvNets still reached Dice values comparable to mid-ranking Transformers, including MA-Net Inc-v4 and U-Net++ Inc-v4.

Mean test root-length correlation was higher for Transformers than ConvNets (0.952 vs 0.948; $p = 0.198$; Table 1), though this difference was not statistically significant. Overlap is visible in the per-model distributions (Fig. 1, middle). SegFormer B1 had the highest root-length correlation (Additional file 1).

Mean test root-diameter correlation was higher for Transformers than ConvNets (0.861 vs 0.848; $p = 0.054$; Table 1), though this difference was not statistically significant. The top-ranked models for diameter correlation were predominantly Transformers: SegFormer B2, M2F Swin-S, and SegFormer B3 (Additional file 2).

**Table 1:** Summary of architecture-family differences (Transformers vs ConvNets) across metrics

Metric	Transformer mean	ConvNet mean	$\Delta$ (T–C)	*p* (T vs C)
Dice (test)	0.679	0.659	+ 0.021	0.00299
Length *r* (test)	0.952	0.948	+ 0.004	0.198
Diameter *r* (test)	0.861	0.848	+ 0.014	0.0542

### Pre-training improves performance

**Fig. 2:**
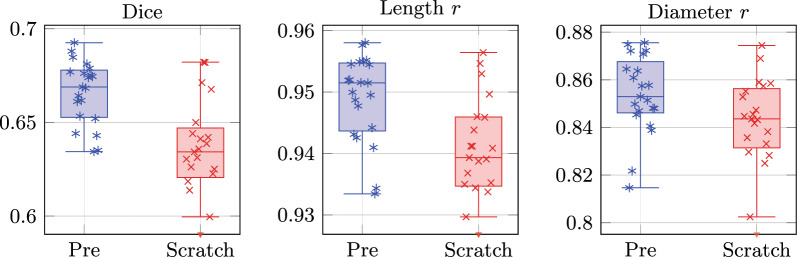
Per-model test performance by pretraining. Left: Dice. Middle: Length *r*. Right: Diameter *r*. *r* = Pearson correlation coefficient. Scratch-trained SAM2 models excluded as outliers

Pre-trained models had significantly higher mean test Dice than models trained from scratch (0.666 vs 0.623; $p = 6.6 \times 10^{-10}$; Table 2; Fig. 2). Pre-trained models also had significantly higher root-length correlation (0.949 vs 0.907; $p = 3.1 \times 10^{-4}$) and root-diameter correlation (0.854 vs 0.828; $p = 4.6 \times 10^{-3}$). The highest-performing models, including MobileSAM ViT-T, M2F Swin-S, and SegFormer B2, were pre-trained, while the lowest-performing were trained from scratch (Table 3; Additional files 1–2).

**Table 2:** Summary of pretraining effects (Pretrained vs Scratch) across metrics

Metric	Pretrained	Scratch	$\Delta$	*p* (Pre vs Scr)	*p* ($\Delta _\textrm{T}$ vs $\Delta _\textrm{C}$)
Dice (test)	0.666	0.623	+0.043	6.62e−10	
Transformer	0.678	0.606	+0.072	3.64e−07	0.000367
ConvNet	0.657	0.636	+0.021	5.4e−05	
Length *r* (test)	0.949	0.907	+0.042	0.00031	
Diameter *r* (test)	0.854	0.828	+0.026	0.00464	

Architecture-specific breakdown shows Transformers benefit significantly more from pre-training than ConvNets; *p* ($\Delta _\textrm{T}$ vs $\Delta _\textrm{C}$) tests whether this difference in benefit is significant

The benefit of pre-training differed by architecture type. Transformer models had a mean Dice improvement of +0.072 from pre-training, compared to +0.021 for ConvNets ($p = 3.7 \times 10^{-4}$; Table 2).

### Model rankings

**Fig. 3:**
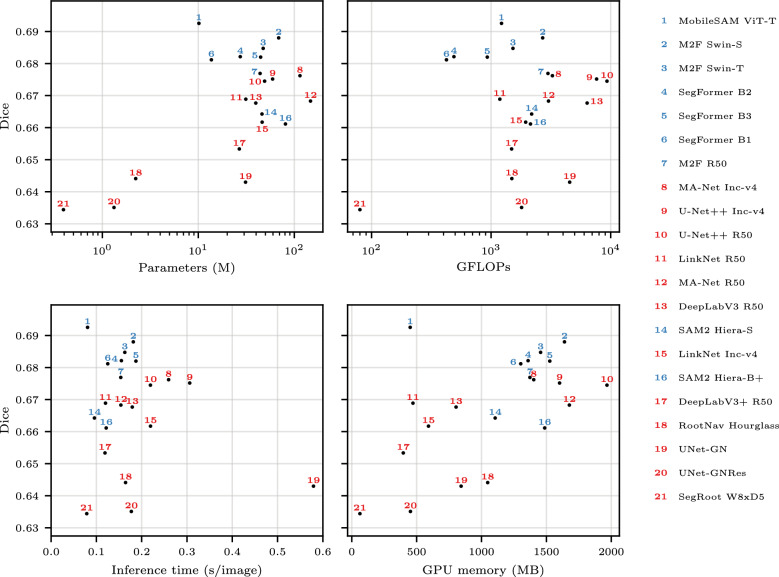
Test Dice vs computational cost. Each panel shows Dice against a different cost metric: parameters, GFLOPs, inference time per image (seconds), and GPU memory (MB). Models are numbered by Dice rank (1 = highest Dice, matching Table 3). Blue = Transformer, red = ConvNet. Inference time and GPU memory were measured end-to-end (tiling, forward passes, stitching) on actual test images at each model’s native patch size, averaged across datasets

Model rankings by test Dice are shown in Table 3. MobileSAM ViT-T had the highest test Dice (0.693), followed by M2F Swin-S (0.688) and M2F Swin-T (0.685). Rankings by root-length and root-diameter correlation are provided in Additional files 1 and 2. Some models ranked highly for Dice but lower for geometric agreement. For example, MobileSAM led in Dice, but SegFormer B1 had the highest root-length correlation. Computational cost metrics including parameters, GFLOPs, inference time, and GPU memory are reported in Table 3 and Fig. 3.

**Table 3:** Validation and test performance (mean Dice) of each model, averaged across datasets; models are ranked by test Dice

Rank	Model	Arch	Val Dice	Test Dice	LR	Pre	Params (M)	GFLOPs	Time (s)	GPU (MB)
1	MobileSAM ViT-T	T	0.7035	0.6925	1e-04	$\checkmark$	10.1	1222	0.081	450
2	M2F Swin-S	T	0.6985	0.6880	1e-04	$\checkmark$	68.7	2698	0.181	1640
3	M2F Swin-T	T	0.7007	0.6847	1e-04	$\checkmark$	47.4	1523	0.163	1455
4	SegFormer B2	T	0.6977	0.6821	1e-04		27.3	489	0.155	1359
5	SegFormer B3	T	0.6942	0.6820	1e-04		44.6	929	0.187	1526
6	SegFormer B1	T	0.6912	0.6812	1e-04	$\checkmark$	13.7	423	0.125	1302
7	M2F R50	T	0.6951	0.6769	1e-04	$\checkmark$	44.0	2989	0.154	1373
8	MA-Net Inc-v4	C	0.6869	0.6762	1e-04	$\checkmark$	114.6	3256	0.259	1403
9	U-Net++ Inc-v4	C	0.6825	0.6752	1e-04	$\checkmark$	59.4	7627	0.306	1602
10	U-Net++ R50	C	0.6879	0.6745	1e-04	$\checkmark$	49.0	9313	0.219	1967
11	LinkNet R50	C	0.6760	0.6689	1e-04	$\checkmark$	31.2	1183	0.120	472
12	MA-Net R50	C	0.6884	0.6683	1e-04	$\checkmark$	147.5	3021	0.154	1677
13	DeepLabV3 R50	C	0.6837	0.6677	1e-04		39.7	6355	0.179	804
14	SAM2 Hiera-S	T	0.6721	0.6643	1e-04	$\checkmark$	46.1	2183	0.096	1105
15	LinkNet Inc-v4	C	0.6783	0.6617	1e-03	$\checkmark$	46.2	1947	0.220	591
16	SAM2 Hiera-B+	T	0.6661	0.6611	1e-04	$\checkmark$	80.9	2135	0.122	1487
17	DeepLabV3+ R50	C	0.6804	0.6534	1e-04	$\checkmark$	26.7	1485	0.119	397
18	RootNav Hourglass	C	0.6531	0.6441	1e-03	$\checkmark$	2.2	1491	0.164	1048
19	UNet-GN	C	0.6536	0.6430	1e-04	$\checkmark$	31.0	4542	0.579	843
20	UNet-GNRes	C	0.6444	0.6351	1e-03	$\checkmark$	1.3	1800	0.177	452
21	SegRoot W8xD5	C	0.6450	0.6344	1e-03	$\checkmark$	0.4	80	0.079	63

Arch = architecture family (T = Transformer, C = ConvNet), LR = learning rate, Pre = pre-trained ($\checkmark$), Params = number of parameters in millions, GFLOPs = billions of floating point operations per forward pass, Time = mean inference time per image in seconds, GPU = GPU memory in MB after inference

### Qualitative results

**Fig. 4:**
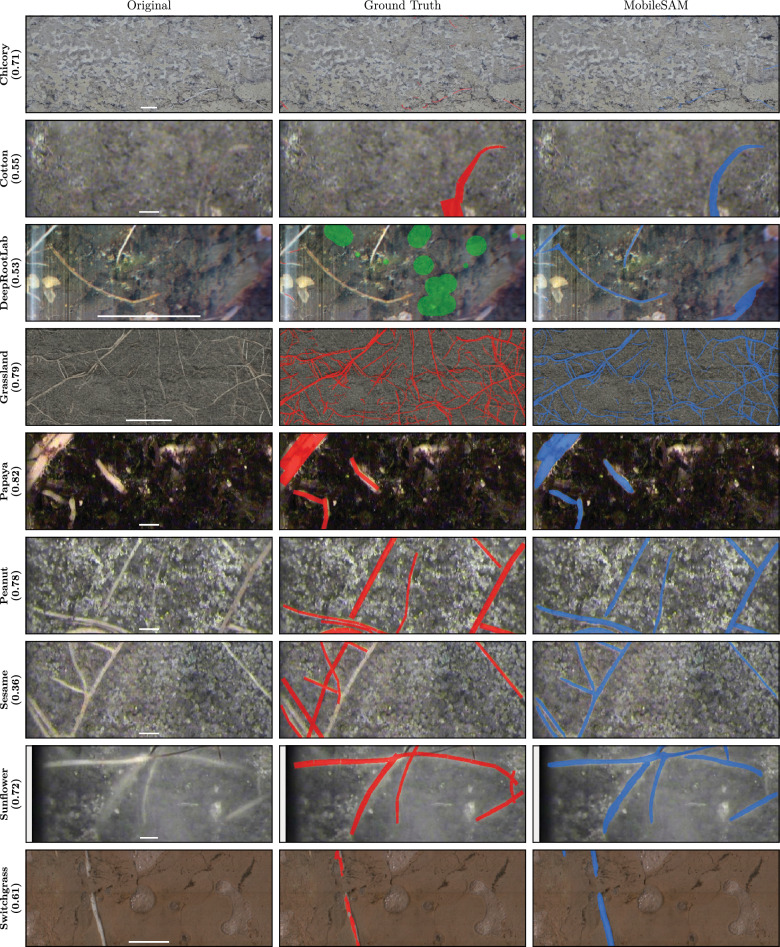
Qualitative comparison of MobileSAM predictions. Each row shows the test image with median Dice for that dataset: original image (left), ground truth overlay in red (middle), and MobileSAM prediction overlay in blue (right). Chicory is cropped to the central 25% for visibility. Scale bars (1 cm) shown in bottom center of original images

Figure 4 shows example segmentations from MobileSAM, the top-ranked model, across all nine datasets. Annotation errors are visible in the Sesame ground truth. In the Papaya example, the model captures root edges more accurately than the manual annotation.

**Fig. 5:**
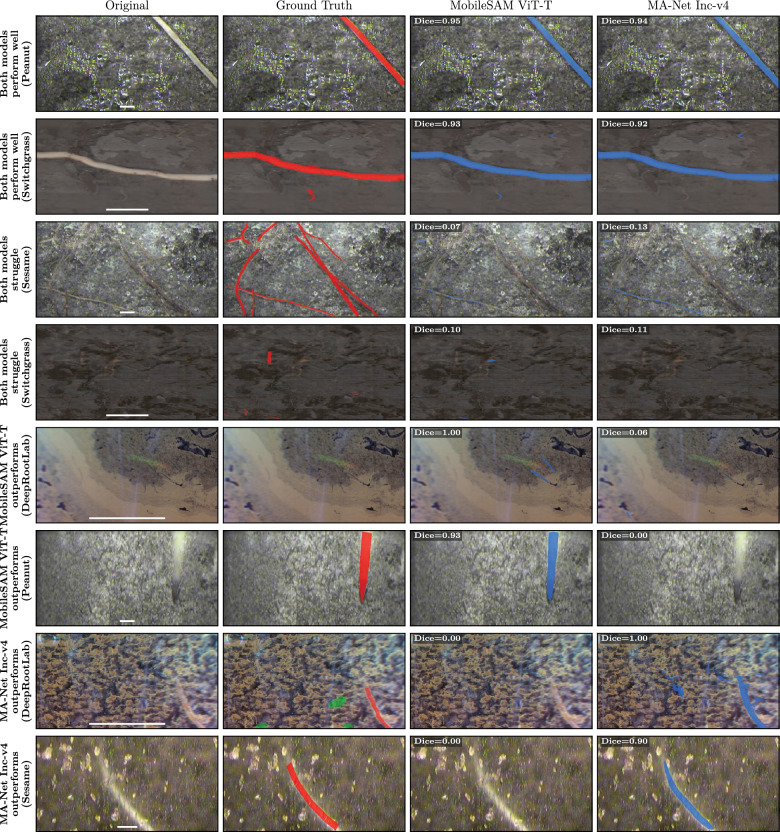
Qualitative comparison of MobileSAM ViT-T (top-ranked Transformer) and MA-Net Inc-v4 (top-ranked ConvNet). Images were selected as: the two with highest mean Dice where both models performed similarly (top), the two with lowest mean Dice where both struggled (second), the two with largest Dice difference favouring MobileSAM (third), and the two with largest Dice difference favouring MA-Net (bottom). Images where both models scored zero were excluded, and the better-performing model was required to have Dice $> 0.5$ for the single-model advantage panels. Ground truth annotations shown in red, model predictions in blue. Per-image Dice shown in each prediction panel. Images are cropped to the region of interest (centered on where models disagree or where roots are present)

To compare the top-ranked Transformer (MobileSAM ViT-T) and top-ranked ConvNet (MA-Net Inc-v4) directly, we computed per-image Dice for both models across 5,603 test images (excluding images where both models scored zero). MobileSAM had higher Dice on 54.0% of images and MA-Net Inc-v4 on 45.9% (Wilcoxon signed-rank test, $p = 6.7 \times 10^{-11}$). At the dataset level, MobileSAM had higher mean Dice on 5 of 9 datasets (mean difference 0.016). Figure 5 shows representative examples where both models perform well, where both struggle, where MobileSAM outperforms, and where MA-Net outperforms. No consistent visual pattern was identified for when one architecture outperforms the other, consistent with the low Architecture Family $\times$ Dataset interaction (0.3%; Section Dataset variation exceeds model variation).

### Dataset variation exceeds model variation

**Fig. 6:**
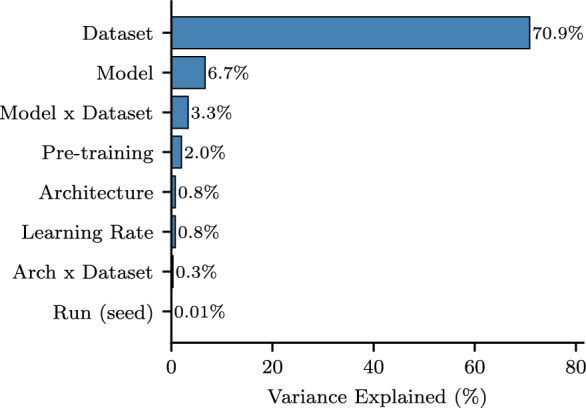
Proportion of variance in test Dice ($\eta ^2$) attributable to each experimental factor. Dataset choice alone accounts for 70.9% of performance variance, far exceeding model architecture, pre-training strategy, or hyperparameter selection

The variation in mean Dice scores across datasets (Table 4) was larger than the variation across model architectures (Table 3). Dataset choice explains 70.9% of variance in test Dice, far exceeding model choice (6.7%), pre-training (2.0%), architecture family (0.8%), learning rate (0.8%), and random seed (0.01%; Fig. 6). Model $\times$ Dataset interaction explained only 3.3% of Dice variance, and Architecture Family $\times$ Dataset interaction only 0.3%, indicating no meaningful architecture-dataset specificity. Transformers had higher mean Dice than ConvNets on 8 of 9 datasets.

**Table 4:** Mean test performance per dataset, averaged across the selected models used in Table 3

Dataset	Mean test dice
Papaya	0.8238
Grassland	0.7739
Peanut	0.7730
Chicory	0.6986
Sunflower	0.6957
Switchgrass	0.6911
Cotton	0.5965
DeepRootLab	0.4916
Sesame	0.4679

### Diameter distribution

**Fig. 7:**
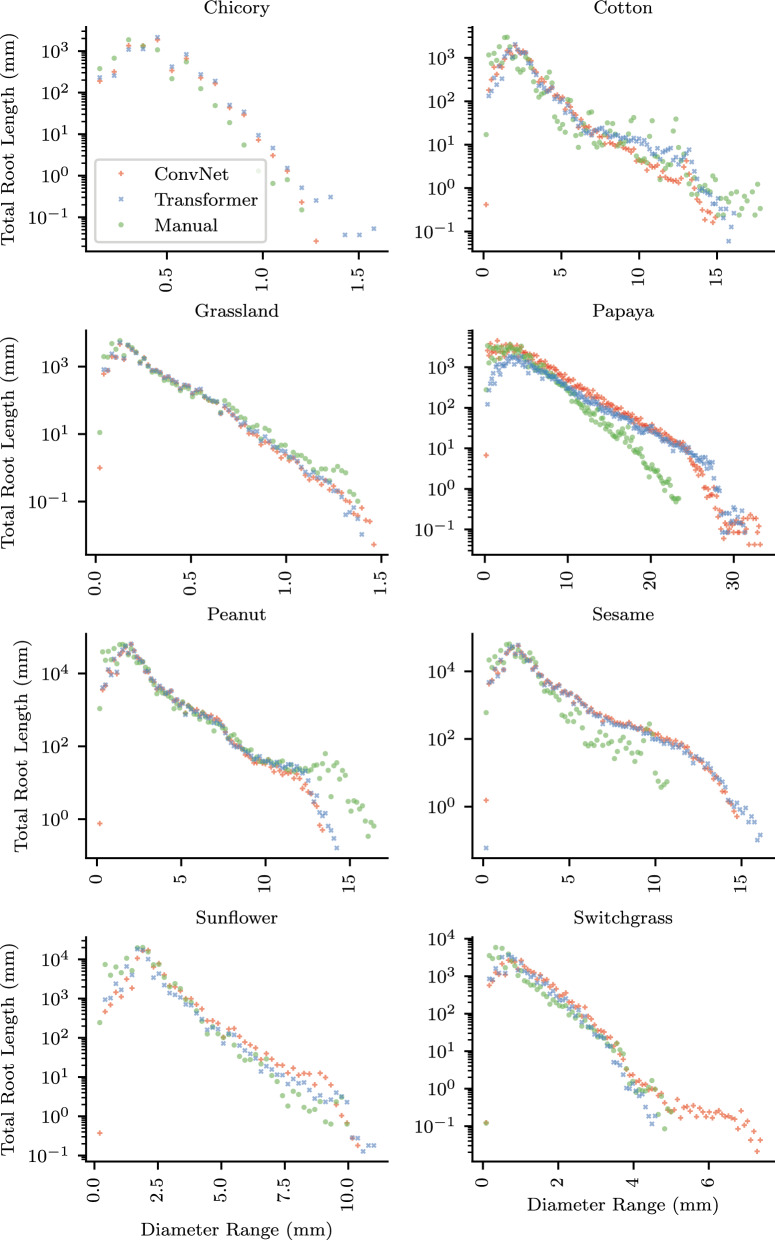
Diameter distribution per dataset. Each panel shows the percentage of total root length at each diameter bin (1-pixel resolution, converted to mm using dataset-specific image resolution) for manual annotations, ConvNets (mean of top 4 from Table 3), and Transformers (mean of top 4 from Table 3). The log scale reveals the exponential decay of root length with increasing diameter

**Fig. 8:**
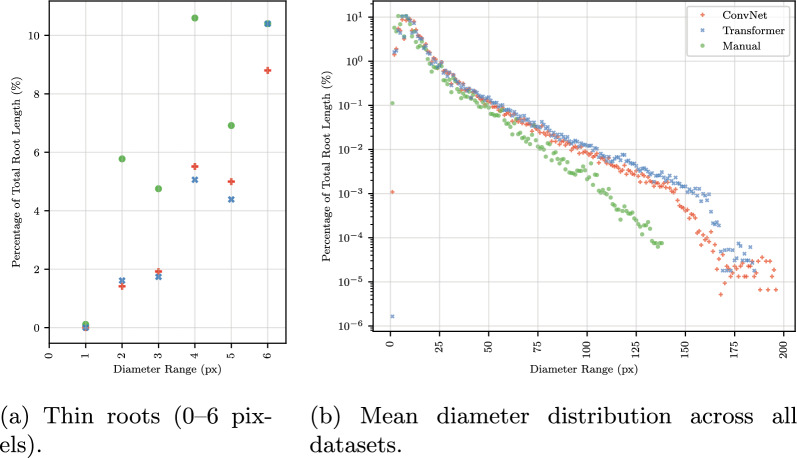
Diameter distributions averaged across datasets, showing manual annotations, ConvNets (mean of top 4 from Table 3), and Transformers (mean of top 4 from Table 3).** a** Thin roots showing underestimation by models on average.** b** Full range with log scale

**Fig. 9:**
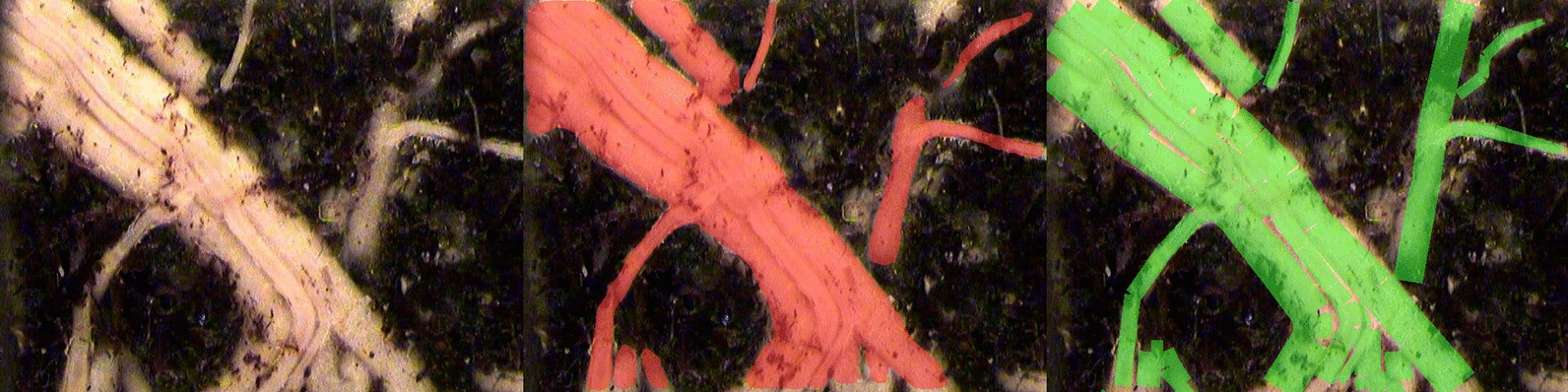
Root merging inflates diameter measurements. The image with the largest discrepancy between predicted and annotated maximum root diameter in the Papaya dataset is shown: original image (left), MobileSAM prediction in red (center), and manual annotation in green (right). Adjacent roots that run in parallel are segmented as a single wide region by the model, causing RhizoVision Explorer to measure them as thick roots (predicted maximum diameter: 167 px) even though individual roots are thinner (annotated maximum diameter: 66 px). This root merging effect explains the model predictions at high diameter bins in Fig. 7, particularly in Papaya, Sesame, and Switchgrass

The distribution of root length across diameter bins showed how models capture root morphology at different scales (Figs. 7, 8b). Figure 7 presents per-dataset diameter distributions in mm. The cross-dataset average (Fig. 8) uses pixel units because dataset resolutions span an eleven-fold range (4.7 to 51.2 px/mm; Table 5), precluding averaging in a common physical scale. For medium and large diameter roots, both ConvNet and Transformer architectures closely tracked manual annotations, following the characteristic exponential decay from the peak at approximately 5–10 pixels (Fig. 8b). For the thinnest roots, however, models on average underestimated root length. At diameter bins below 5 pixels, manual annotations consistently attributed a higher percentage of total root length than either architecture (Fig. 8a). At 2 pixels diameter, manual annotations showed 4.7% of root length compared to 1.4–1.7% for models; at 4 pixels, 8.2% compared to approximately 5%. Both ConvNet and Transformer models underestimated thin roots, either by failing to detect them or by predicting thicker diameters than annotated. This limitation was consistent across datasets (Fig. −7) and is important for applications where accurate measurement of fine root structures is critical. At the thick end of the distribution, models predicted root length at diameter bins beyond the maximum annotated diameter, particularly in Papaya, Sesame, and Switchgrass (Fig. 7). Inspection of the most extreme cases revealed that root merging contributes to this discrepancy: when adjacent roots run in parallel, models segment them as a single wide region, inflating the measured diameter (Fig. 9).

**Fig. 10:**
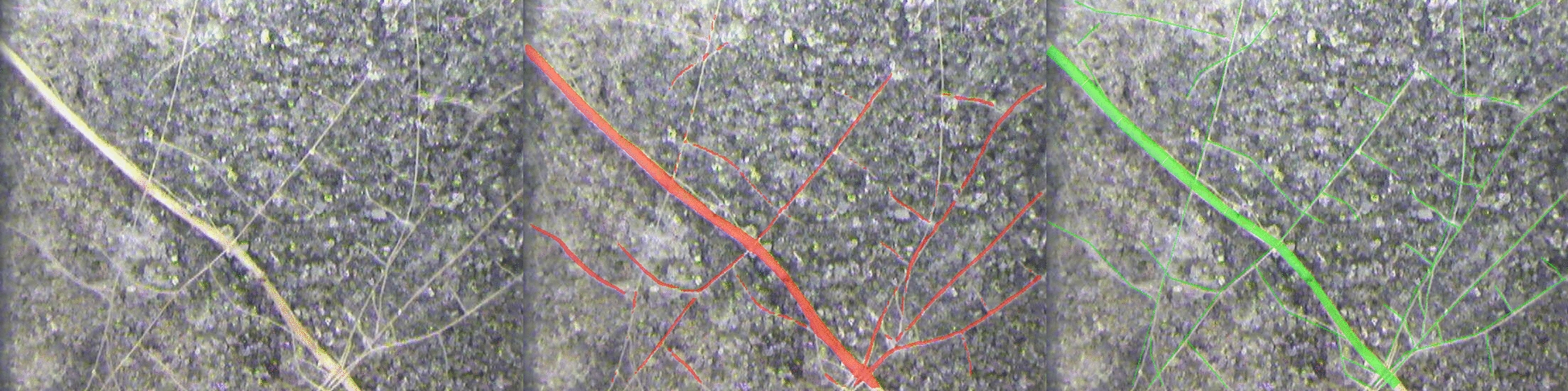
Thin roots missed by the model. The Peanut image with the largest thin-root length discrepancy (diameter bin 2, $-2993$ px) is shown: original image (left), MobileSAM prediction in red (center), and manual annotation in green (right). Several fine lateral roots visible in the annotation are not captured by MobileSAM

**Fig. 11:**
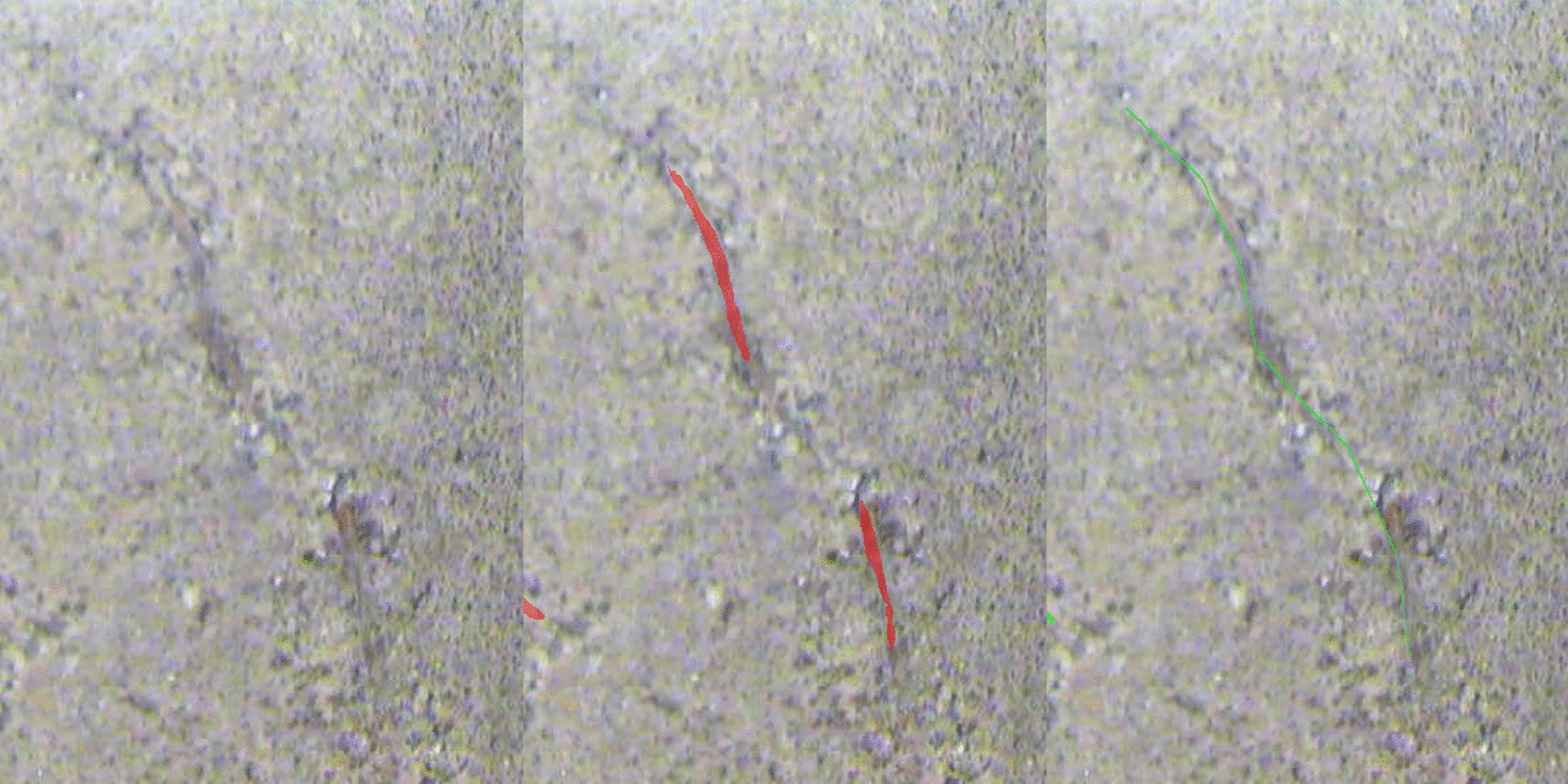
Annotation error in Cotton: roots traced too thin. Top half of the Cotton image with the largest “predicted too thick” discrepancy in diameter bin 2 ($-353$ px): original image (left), MobileSAM prediction in red (center), and manual annotation in green (right). The model under-segments some root length but captures root thickness more accurately than the annotation, which traces roots thinner than they appear in the original image

Thin root length underestimation fell into three dominant error types: RVE measurement artifacts from annotation corners (31%), roots missed entirely by the model (24%; Fig. 10), and roots where the model segmentation was wider than the annotation (42%). Wider predictions occurred in all datasets, and missed roots in all datasets except Chicory. However, wider predictions do not always indicate a model error: in some cases the annotation was traced thinner than the actual root visible in the scan (Fig. 11), indicating that annotation quality also contributes to thin root discrepancies.

## Discussion

### Transformer architectures outperform ConvNets

The consistent direction of Transformer improvement across Dice, root-length correlation, and root-diameter correlation supports H1, even though root-length and root-diameter correlation were not statistically significant ($p = 0.198$ and $p = 0.054$ respectively). This finding aligns with broader evidence from dense prediction tasks, where Vision Transformers produce more robust features than CNNs while relying less on texture cues [13]. A systematic review of 36 medical imaging studies similarly found that Transformer-based models exhibit superior performance compared to CNNs, though with greater dependence on pre-training [14]. Our results extend these findings to root segmentation. One possible explanation is that the elongated, branching structure of roots benefits from Transformers’ self-attention mechanism, which integrates image-wide context from early layers in the network, unlike ConvNets which build up context gradually through successive layers. Other factors such as more effective transfer learning across large domain gaps may also contribute. The Transformer advantage was consistent across 8 of 9 datasets, with the largest improvement on DeepRootLab, suggesting the findings generalize to challenging field conditions. Explainability methods may provide further mechanistic insight into why Transformers outperform ConvNets for root segmentation.

Both SAM2 and MobileSAM were fine-tuned with a null prompt. MobileSAM achieved the highest Dice overall, while SAM2 was the least accurate Transformer. One possible explanation is that SAM2 may be more dependent on informative prompts, while MobileSAM performs well fine-tuned without them. Unlike MobileSAM, which is designed purely for single-image segmentation, SAM2 was designed to handle both image and video segmentation. Sengupta et al. [15] found SAM2 performance lacking compared to SAM in lower contrast CT and ultrasound images, suggesting SAM2 may struggle in some single-image segmentation settings.

### Pre-training benefits generalization

The consistent improvement from pre-training across all metrics shows that transfer learning provides useful initialisation despite the domain shift between pre-training datasets and root imagery, supporting H2. Transfer learning is widely adopted in agricultural imaging [16], with reported benefits including faster convergence and reduced training time [17]. Our results confirm a modest but significant accuracy improvement (+0.043 Dice), though models trained from scratch still achieved reasonable performance.

Jeeveswaran et al. [13] and Takahashi et al. [14] claimed that Transformers are more data-hungry architectures, requiring larger datasets for effective training compared to ConvNets. The comparatively weak performance we observed for Transformers trained from scratch supports these claims, with a mean Dice of 0.606 compared to 0.636 for ConvNets trained from scratch. Zhou et al. [18] hypothesised that pre-trained Transformers generalise better under large domain gaps, which may also contribute to the significantly greater benefit Transformers gained from pre-training compared to ConvNets (+0.072 vs +0.021 Dice; Table 2). Our pre-trained Transformer models used readily available weights from ImageNet, COCO, and Cityscapes, none of which contain root imagery, representing a large domain gap.

### Diameter distribution and thin root detection

Both Transformers and ConvNets underestimated thin roots, attributing less root length to the smallest diameter bins than manual annotations (Fig. 8a). This parallels the “extremely small target” problem in medical image segmentation, where structures occupying few pixels can be ignored by networks trained with pixel-wise losses [19]. Fine root structures also present known challenges across root phenotyping methods due to resolution limits [1]. Our manual inspection of the largest thin root discrepancies showed that this reflects both genuine model limitations, roots missed entirely (Fig. 10), and annotation inconsistency, where annotators traced roots thinner than they appear in the original image (Fig. 11). The RVE measurement artifacts observed in 31% of inspected images arose from sharp corners in annotation masks produced by the annotation tool used for some datasets, and can likely be mitigated in practice using RVE’s built-in root pruning option, which removes short skeleton segments.

### Model efficiency and practical recommendations

For practitioners with constrained computational resources, pre-trained MobileSAM offers the best trade-off, achieving the highest Dice with low inference time (0.081 s/image), few parameters (10.1M), and modest GPU memory (450 MB; Fig. 3; Table 3). For applications where computational cost is the primary constraint and the segmentation task is relatively straightforward, SegRoot W8xD5 offers the lowest parameters (0.4M), FLOPs (80 GFLOPs), and GPU memory (63 MB) of any evaluated model.

### Sources of variance

Dataset choice dominated performance variance (70.9%) compared to model choice (6.7%). Dataset-related factors, including inherent task difficulty, image quality, annotation quality, and dataset size, caused larger differences in segmentation performance than model architecture choice. Datasets can differ in inherent task difficulty due to factors such as root appearance, soil background complexity and artifacts, and species. The negligible effect of random seed (0.01%) shows high reproducibility. Given that dataset factors dominate performance variance, our results suggest practitioners should prioritize data curation over architecture selection. What constitutes a sufficient Dice for practical use will depend on the features of interest and dataset.

Annotation quality varied across datasets and likely contributed to the measured performance differences. In the Sesame dataset, we observed annotation errors including misaligned masks and incomplete root tracing (Fig. 4), which contributed to lower Dice scores. For DeepRootLab, the corrective annotation approach likely contributes to lower Dice, as we only measure performance on harder regions of the image where models are more likely to have errors. Because these annotations were created by correcting errors, they may disproportionately capture the differences between stronger and weaker models, which could explain why DeepRootLab showed the widest spread in per-model Dice (range 0.33 vs $\le$0.08 for all other datasets; Additional files 5–6). Despite this, the overall model ranking was stable: removing DeepRootLab and averaging the remaining eight datasets yielded a ranking highly correlated with the full nine-dataset ranking (Spearman $\rho = 0.92$). Additionally, this dataset spans eleven species imaged over multiple years under field conditions, introducing challenging variation in root appearance and age.

Manual inspection of thin root discrepancies further showed that some cases where the model predicted thicker roots were in fact annotation errors, where roots were traced thinner than they appear in the original image (Fig. 11). These annotation issues highlight a broader challenge: when models segment roots more accurately than annotators, standard metrics penalize the model.

### Limitations and future work

Pre-trained weights were sourced from ImageNet, COCO, Cityscapes, or model-specific datasets, reflecting the practical reality that researchers typically fine-tune from publicly available checkpoints. Because Transformer and ConvNet models used different pre-training sources (Table 6), the larger improvement observed for Transformers may be partially due to differences in pre-training source rather than architecture alone. The consistent advantage of pre-training across this heterogeneous set of sources shows that H2 holds regardless of the specific pre-training dataset used. Domain-specific pre-training on root imagery could yield further gains. The discrepancy between model and annotation at thin root diameters (Fig. 8a), which reflects both genuine model limitations and annotation inconsistencies (Figs. 10 and 11), indicates that improvements in both segmentation models and annotation protocols would benefit root phenotyping applications.

Transformer models used larger patch sizes (1024 pixels) than ConvNets (572–576 pixels), providing more spatial context per forward pass. To assess the effect of patch size, we trained the best Transformer (MobileSAM) at 576 pixels and the best ConvNet (MA-Net Inc-v4) at 1024 pixels. Even with the smaller patch size, MobileSAM still outperformed MA-Net with the larger patch size (mean Dice 0.684 vs 0.679), indicating that the Transformer advantage is not explained by patch size (Additional file 8).

## Conclusions

We evaluated 21 deep learning architectures across nine root image datasets (1,511 training runs). Transformer architectures had significantly higher Dice than ConvNets ($p = 3.0 \times 10^{-3}$). Root-diameter and root-length correlation were also higher for Transformers, though the differences were not statistically significant ($p = 0.054$ and $p = 0.198$ respectively). MobileSAM achieved the highest Dice (0.693). Pre-training improved performance ($p = 6.6 \times 10^{-10}$), particularly for Transformers (+0.072 vs +0.021 Dice, $p = 3.7 \times 10^{-4}$). Dataset choice explained 70.9% of performance variance, far exceeding model choice (6.7%). For practitioners, investing in training data quality and quantity matters more than architecture selection. Among models, pre-trained MobileSAM offers strong accuracy with low computational cost.

## Methods

### Datasets

We evaluated model performance on nine publicly available datasets spanning diverse species, root appearances, and imaging conditions. All datasets are open access, supporting reproducibility. We selected these datasets to represent variation in imaging modality, scale, and annotation density. The datasets are described below with references to the original publications. Example images are shown in Fig. 4; dataset splits, image resolutions, and DPI are provided in Table 5.

*DeepRootLab:* This dataset includes images of eleven species, collected and annotated as part of the Five Seasons project [20]. The species are: intermediate wheatgrass (*Thinopyrum intermedium*), perennial lupine (*Lupinus perennis*), chicory (*Cichorium intybus*), dyers woad (*Istatis tinctoria*), mugwort (*Artemisia vulgaris*), rosinweed (*Silphium perfoliatum*), comfrey (*Symphytum officinale*), curly dock (*Rumex crispus*), tall fescue (*Festuca arundinacea*), winter rye (*Secale cereale*), and winter wheat (*Triticum aestivum*). Annotations represent corrective edits rather than complete root traces; consequently, this dataset is excluded from root-length and root-diameter correlation analyses. Images are available from https://zenodo.org/records/15213661.

*Grassland:* Collected in alpine grasslands and released as part of a study on root growth and soil function [2], this dataset captures natural root-soil interactions under field conditions. Images are available from https://figshare.com/ndownloader/articles/20440497/versions/2.

*Chicory:* We used the densely annotated subset of the chicory dataset introduced in [5] and published in [21]. Images are available from https://zenodo.org/records/3527713.

*PRMI Collection:* These six datasets, papaya (*Carica papaya*), peanut (*Arachis hypogaea*), sesame (*Sesamum indicum*), sunflower (*Helianthus annuus*), cotton (*Gossypium hirsutum*), and switchgrass (*Panicum virgatum*), were released as part of the PRMI benchmark for minirhizotron root imaging [22]. Each dataset corresponds to a different crop species and imaging context. Images are available from https://gatorsense.github.io/PRMI/.

We used the original dataset splits where available (PRMI benchmark, Grassland, Chicory). For DeepRootLab, which did not have an established annotated train/val/test split, we split images 60/20/20 into training, validation, and test sets, to approximate what is typically used in machine learning.

**Table 5:** Number of images in each dataset split, with image resolution and DPI

Dataset	Train	Val	Test	Total	DPI	px/mm	Image Size (px)
Chicory	29	9	10	48	338	13.3	3991$\times$1842
Cotton	1271	564	577	2412	150	5.9	736$\times$552
DeepRootLab	262	86	90	438	1300	51.2	1224$\times$1024
Grassland	71	21	16	108	1200	47.2	2550$\times$2196
Papaya	282	131	133	546	150	5.9	736$\times$552
Peanut	11,485	3347	4793	19,625	150	5.9	736$\times$552
Sesame	8637	2625	3084	14,346	150	5.9	736$\times$552
Sunflower	2211	722	967	3900	120	4.7	640$\times$480
Switchgrass	2,647	665	600	3912	300	11.8	510$\times$720
Total	**26,895**	**8170**	**10,270**	**45,335**			

Bold values indicate the optional formatting to distinguish the totals from individual dataset rows

### Neural network architectures

The evaluated architectures (Table 6) include both convolutional and Transformer models, with and without pre-trained weights. Architectures were selected because they are conveniently available, open source, and have publicly available pre-trained weights. U-Net, DeepLabV3+, RootNav 2.0, and SegRoot were of particular interest as these have been previously evaluated for root segmentation.

**Table 6:** Evaluated segmentation architectures, encoders, and pre-training sources

Architecture	Encoder	Pre-training	Type
U-Net++	ResNet50 [23]	ImageNet [24]	ConvNet
	InceptionV4 [25]		
LinkNet	ResNet50	ImageNet	ConvNet
	InceptionV4		
MAnet	ResNet50	ImageNet	ConvNet
	InceptionV4		
SegRoot	w8d5 [26]	SegRoot	ConvNet
Mask2Former	ResNet50	COCO [27]	Transformer
	Swin-Tiny	COCO [27]	
	Swin-Small [28]	COCO [27]	
SegFormer	mit-b1	Cityscapes [29]	Transformer
	mit-b2	Cityscapes [29]	
	mit-b3 [30]	Cityscapes [29]	
UNetGN	GroupNorm [5]	Chicory$^*$	ConvNet
UNetGNRes	GroupNorm + res. [31]	Chicory$^*$	ConvNet
DeepLabV3/V3+	DeepLabV3 (ResNet50)	ImageNet	ConvNet
	DeepLabV3+ (ResNet50)		
RootNav 2.0	Stacked hourglass [32]	RootNav	ConvNet
MobileSAM	ViT-Tiny [33]	SA-1B [34]	Transformer
SAM2	Hiera-Small	SA-V [35]	Transformer
	Hiera-Base+		

$^*$Excluded from pre-trained evaluation on Chicory to prevent data leakage

#### Convolutional architectures

 These include encoder-decoder models widely used in biomedical and plant image analysis, as well as several lightweight or attention-augmented variants:

- UNetGN and UNetGNRes, both based on the original U-Net architecture [6], were adapted for root segmentation by replacing BatchNorm with GroupNorm to better accommodate small batch sizes. UNetGN was introduced in [5], while UNetGNRes is a residual variant with reduced parameters, developed as part of RootPainter [31].
- U-Net++ [36], which enhances the U-Net architecture with nested and dense skip connections. We used the implementation from Segmentation Models Pytorch [37].
- DeepLabV3 and DeepLabV3+ [38, 39], encoder-decoder models using dilated convolutions and ResNet-50 backbones. The implementation for DeepLabV3 was taken from TorchVision [40], while the DeepLabV3+ implementation was from Segmentation Models Pytorch [37].
- LinkNet [41] and MAnet [42], offering residual or attention-based improvements over basic encoder-decoder structures. The implementations were taken from Segmentation Models Pytorch [37].
- RootNav 2.0 [43], a deep hourglass-style network for root system segmentation.
- SegRoot [26], a compact convolutional architecture tailored for fine root segmentation.

#### Transformer architectures

 These models incorporate self-attention mechanisms and hierarchical token processing for segmentation:

- SegFormer [30], a lightweight transformer architecture with multi-scale hierarchical encoding and efficient MLP decoding. We used the implementation from Segmentation Models Pytorch [37], evaluated using MIT-B1, MIT-B2 and MIT-B3 backbones.
- Mask2Former [44], a unified architecture for panoptic and semantic segmentation, tested with ResNet-50, Swin-Tiny, and Swin-Small backbones.
- MobileSAM [45], a mobile-optimized adaptation of the Segment Anything model, using a ViT-Tiny backbone.
- SAM2 [35], the second generation Segment Anything model for images and video, tested with Hiera-Small and Hiera-Base+ backbones.

### Pre-training

Models were evaluated with pre-trained weights and trained from scratch with random initialisation. Pre-training sources are listed in Table 6.

### Training procedure

Each model was trained with two learning rates (0.001 and 0.0001), both with pre-trained weights and from scratch. Each of these four configurations was trained twice with different random seeds, yielding 8 training runs per model per dataset. Across 21 models and 9 datasets, this resulted in 1,511 training runs. One run failed to converge (MobileSAM ViT-T on DeepRootLab, from scratch at a learning rate of 0.001), never exceeding 0.0 validation Dice.

Training used the AdamW optimiser with 16-bit mixed precision. Each run was limited to a maximum of 2 h. A warm-up schedule of 2 epochs was applied, followed by early stopping if validation Dice did not improve for 20 epochs. The loss function combined Dice loss and cross-entropy, which has proven effective for class-imbalanced root datasets [5]. To mitigate class imbalance, batches containing no root annotations were excluded.

Patch sizes were selected based on architecture requirements: 572 pixels for UNet-GN, 576 pixels for models requiring dimensions divisible by 64, and 1024 pixels for transformer-based architectures. Batch sizes were set to the largest value in {4, 8, 16} that fit in GPU memory.

Data augmentation was applied on the fly during training and included colour jitter (66% probability), greyscale conversion (5%), pixel inversion (2.5%), random rotation (±15$^\circ$, 33%), perspective distortion (33%), horizontal flipping (50%), and elastic deformation ($\alpha$=75, 33%).

All training was performed on two machines with identical hardware, each equipped with an AMD Ryzen 9 7950X CPU, 32GB RAM, and an NVIDIA RTX 4090 GPU with 24GB VRAM. Computational cost was quantified in billions of floating point operations (GFLOPs) per forward pass, computed using the calflops library [46], and number of parameters. Inference time per image and GPU memory were measured end-to-end (tiling, forward passes, stitching) on actual test images at each model’s native patch size, averaged across datasets. GPU memory was recorded as torch.cuda.memory_reserved() after inference. Training code is available at https://github.com/sotlampr/seg.

### Configuration selection

To prevent overfitting to the test set, model selection used a two-stage procedure based on validation performance:

#### Replicate selection

 For each combination of model, dataset, learning rate, and pre-training, the replicate with the highest validation Dice was retained, along with its paired test result.

#### Hyperparameter selection

 For each model, the configuration (learning rate, pre-training) with the highest mean validation Dice across all datasets was selected.

All reported test metrics use the configuration selected by this procedure.

For pre-trained vs scratch comparisons, configuration selection was performed separately within each condition: for each model, the best learning rate was selected independently for pre-trained runs and for scratch runs, both based on mean validation Dice across datasets. This gave paired observations for each model, one pre-trained and one trained from scratch.

### Trait extraction

Root morphological traits were extracted from both predicted segmentations and ground-truth annotations using a fork of RhizoVision Explorer (RVE) [47] with bug fixes and headless functionality (see Additional file 7), parallelized with GNU Parallel [48]. For each segmentation mask, we computed total root length and mean root diameter. These traits allow evaluation beyond pixel-level accuracy, assessing whether models produce segmentations that yield biologically meaningful measurements. Total root length is relevant for rooting density and specific root length, a widely measured functional trait, while mean diameter captures root thickness which relates to root development and function.

Additionally, we configured RVE to compute root length binned by diameter in 1-pixel increments, enabling analysis of how root length is distributed across different root diameters. This diameter distribution reveals whether models accurately capture the full range of root sizes, particularly thin roots which are challenging to segment.

### Evaluation metrics

#### Dice coefficient

 Primary segmentation quality metric, computed as the harmonic mean of precision and recall between predicted and ground-truth masks.

#### Root-length correlation

 Pearson correlation coefficient between predicted and ground-truth total root length, measuring agreement in overall root quantity estimation.

#### Root-diameter correlation

 Pearson correlation coefficient between predicted and ground-truth mean root diameter, measuring agreement in root thickness estimation.

### Statistical analysis

To compare Transformer and ConvNet architectures (a between-model comparison), we averaged each model’s metric across datasets and applied an independent two-sample *t*-test on model means ($n = 9$ Transformer, $n = 12$ ConvNet) with a two-sided alternative. For pre-training comparisons and the architecture-by-pre-training interaction (within-model contrasts across datasets), we used linear mixed effects models with dataset and model as random intercepts to account for the repeated-measures structure. All tests used two-sided alternatives.

All tests used a significance level of $\alpha = 0.05$. All configuration selection was performed on validation data; all reported metrics are from held-out test sets.

To quantify the contribution of each experimental factor to performance variance, we computed eta-squared ($\eta ^2 = SS_{\text {between}} / SS_{\text {total}}$) independently for each factor: dataset, model, architecture family, pre-training, learning rate, and random seed. Each factor’s variance contribution was assessed separately rather than in a joint model. To assess architecture-dataset specificity, we additionally computed eta-squared for the Model $\times$ Dataset and Architecture Family $\times$ Dataset interaction terms.

### Thin root discrepancy analysis

To understand the sources of thin root discrepancy between model predictions and annotations, we selected the 10 images with the largest root length underestimation per diameter bin (bins 1–6, in 1-pixel increments) for MobileSAM across all eight datasets, or fewer where a bin contained fewer than 10 images, yielding 437 images. Each image was visually inspected and the dominant source of error was identified: an RVE measurement artifact (where annotation corners were falsely detected as thin roots), roots missed entirely by the model, or roots predicted too thick (model segmentation wider than the annotation). Qualitative examples (Figs. 10 and 11) were selected as the images with the largest discrepancy for each error type.

### Use of AI assistants

Claude (Anthropic) was used to assist with code development and manuscript preparation. All AI-generated content was reviewed and verified by the authors.

## Supplementary Information

Supplementary Material 1.

Supplementary Material 2.

## Data Availability

All nine root image datasets used in this study are publicly available. DeepRootLab images are available from Zenodo (https://zenodo.org/records/15213661). Grassland images are available from Figshare (https://figshare.com/ndownloader/articles/20440497/versions/2). Chicory images are available from Zenodo (https://zenodo.org/records/3527713). The six PRMI datasets (Papaya, Peanut, Sesame, Sunflower, Cotton, Switchgrass) are available from https://gatorsense.github.io/PRMI/. Training code is available at https://github.com/sotlampr/seg. The modified RhizoVision Explorer fork used for trait extraction is available at https://github.com/sotlampr/RhizoVisionExplorer.
