# Supplementary material for: A systematic comparison of transformers and ConvNets for root segmentation across nine datasets
Source: Plant Methods. 2026 Apr 20;22:52. doi: 10.1186/s13007-026-01533-6 (PMC13227879; doi:10.1186/s13007-026-01533-6)
Supplement: Supplementary file 1 — Supplementary Material 1. [file 13007_2026_1533_MOESM1_ESM.pdf]

Additional Files: A Systematic Comparison of Transformers and ConvNets for Root Segmentation Across Nine Datasets

Abraham George Smith, Sotiris Lamprinidis, Anand Seethepalli, Larry M. York, Eusun Han, Patrick Möhl, Kyriaki Boulata, Kristian Thorup-Kristensen, Jens Petersen

Additional file 1: Model ranking by root-length correlation

Root length agreement (Pearson correlation r) on the test set, averaged across datasets; configurations are selected using validation Dice, and models are ranked by test r. Arch = architecture family (T = Transformer, C = ConvNet), Pre = pre-trained (✓).

Rank	Model	Arch	Length r (test)	Pre
1	SegFormer B1	T	0.958	✓
2	MA-Net Inc-v4	C	0.958	✓
3	SegFormer B3	T	0.956	
4	MA-Net R50	C	0.955	✓
5	LinkNet Inc-v4	C	0.955	✓
6	M2F Swin-T	T	0.955	✓
7	U-Net++ Inc-v4	C	0.954	✓
8	SegFormer B2	T	0.953	
9	M2F Swin-S	T	0.952	✓
10	M2F R50	T	0.952	✓
11	LinkNet R50	C	0.952	✓
12	MobileSAM ViT-T	T	0.950	✓
13	DeepLabV3 R50	C	0.950	
14	U-Net++ R50	C	0.950	✓
15	SAM2 Hiera-B+	T	0.949	✓
16	DeepLabV3+ R50	C	0.948	✓
17	SegRoot W8xD5	C	0.944	✓
18	RootNav Hourglass	C	0.943	✓
19	SAM2 Hiera-S	T	0.943	✓
20	UNet-GNRes	C	0.934	✓
21	UNet-GN	C	0.933	✓

Additional file 2: Model ranking by root-diameter correlation

Root diameter agreement (Pearson correlation r) on the test set, averaged across datasets; configurations are selected using validation Dice, and models are ranked by test r. Arch = architecture family (T = Transformer, C = ConvNet), Pre = pre-trained (✓).

Rank	Model	Arch	Diameter r (test)	Pre
1	M2F Swin-S	T	0.875	✓
2	SegFormer B3	T	0.874	
3	MobileSAM ViT-T	T	0.872	✓
4	SegFormer B1	T	0.871	✓
5	SegFormer B2	T	0.869	
6	M2F R50	T	0.865	✓
7	LinkNet Inc-v4	C	0.864	✓
8	SAM2 Hiera-B+	T	0.861	✓
9	U-Net++ R50	C	0.858	✓
10	MA-Net R50	C	0.857	✓
11	MA-Net Inc-v4	C	0.853	✓
12	U-Net++ Inc-v4	C	0.851	✓
13	SAM2 Hiera-S	T	0.850	✓
14	UNet-GN	C	0.848	✓
15	UNet-GNRes	C	0.848	✓
16	LinkNet R50	C	0.847	✓
17	DeepLabV3+ R50	C	0.845	✓
18	SegRoot W8xD5	C	0.841	✓
19	DeepLabV3 R50	C	0.838	
20	RootNav Hourglass	C	0.822	✓
21	M2F Swin-T	T	0.815	✓

Additional file 3: Per-dataset test Dice (pre-trained)

Test Dice for each pre-trained model on each dataset. For each model, the best learning rate is selected by mean validation Dice across datasets. Models are ranked by mean test Dice (descending). T = Transformer, C = ConvNet.

Model	Type	LR	Chicory	Cotton	DeepRootLab	Grassland	Papaya	Peanut	Sesame	Sunflower	Switchgrass
MobileSAM ViT-T	T	10^{-4}	0.705	0.626	0.639	0.789	0.833	0.784	0.469	0.706	0.682
M2F Swin-S	T	10^{-4}	0.705	0.620	0.596	0.782	0.832	0.781	0.467	0.684	0.726
M2F Swin-T	T	10^{-4}	0.706	0.615	0.569	0.792	0.837	0.778	0.474	0.675	0.716
SegFormer B1	T	10^{-4}	0.692	0.627	0.529	0.777	0.832	0.778	0.483	0.699	0.715
SegFormer B3	T	10^{-4}	0.693	0.616	0.545	0.768	0.838	0.786	0.464	0.675	0.725
M2F R50	T	10^{-4}	0.707	0.607	0.546	0.772	0.831	0.772	0.467	0.693	0.699
MA-Net Inc-v4	C	10^{-4}	0.708	0.604	0.532	0.781	0.826	0.784	0.462	0.707	0.683
U-Net++ Inc-v4	C	10^{-4}	0.708	0.593	0.509	0.781	0.827	0.782	0.470	0.715	0.691
U-Net++ R50	C	10^{-4}	0.708	0.603	0.503	0.780	0.830	0.777	0.472	0.707	0.690
SegFormer B2	T	10^{-4}	0.692	0.605	0.476	0.779	0.834	0.785	0.467	0.703	0.725
LinkNet R50	C	10^{-4}	0.698	0.586	0.509	0.771	0.825	0.776	0.467	0.703	0.685
MA-Net R50	C	10^{-4}	0.705	0.599	0.480	0.782	0.816	0.769	0.470	0.713	0.681
SAM2 Hiera-S	T	10^{-4}	0.704	0.570	0.459	0.781	0.831	0.773	0.461	0.706	0.692
LinkNet Inc-v4	C	10^{-3}	0.703	0.600	0.414	0.786	0.823	0.768	0.470	0.712	0.678
SAM2 Hiera-B+	T	10^{-4}	0.701	0.581	0.442	0.776	0.830	0.772	0.475	0.682	0.692
DeepLabV3+ R50	C	10^{-4}	0.687	0.598	0.450	0.770	0.825	0.774	0.453	0.657	0.667
DeepLabV3 R50	C	10^{-4}	0.664	0.598	0.454	0.756	0.823	0.774	0.448	0.685	0.667
RootNav Hourglass	C	10^{-3}	0.700	0.545	0.401	0.762	0.809	0.743	0.466	0.686	0.684
UNet-GN	C	10^{-4}	–	0.577	0.403	0.749	0.797	0.770	0.464	0.690	0.692
UNet-GNRes	C	10^{-3}	–	0.575	0.307	0.782	0.810	0.764	0.470	0.700	0.673
SegRoot W8xD5	C	10^{-3}	0.681	0.560	0.367	0.734	0.801	0.738	0.466	0.683	0.680

Additional file 4: Per-dataset test Dice (trained from scratch)

Test Dice for each model trained from scratch on each dataset. For each model, the best learning rate is selected by mean validation Dice across datasets. Models are ranked by mean test Dice (descending). T = Transformer, C = ConvNet.

Model	Type	LR	Chicory	Cotton	DeepRootLab	Grassland	Papaya	Peanut	Sesame	Sunflower	Switchgrass
SegFormer B2	T	10^{-4}	0.687	0.619	0.563	0.770	0.835	0.785	0.469	0.695	0.716
SegFormer B3	T	10^{-4}	0.693	0.628	0.546	0.777	0.825	0.786	0.469	0.705	0.708
SegFormer B1	T	10^{-4}	0.686	0.620	0.501	0.773	0.827	0.781	0.475	0.663	0.716
DeepLabV3 R50	C	10^{-4}	0.674	0.595	0.561	0.757	0.825	0.777	0.463	0.692	0.664
LinkNet Inc-v4	C	10^{-3}	0.703	0.578	0.385	0.778	0.814	0.758	0.466	0.682	0.685
RootNav Hourglass	C	10^{-4}	0.701	0.546	0.378	0.767	0.804	0.751	0.466	0.697	0.686
U-Net++ R50	C	10^{-4}	0.701	0.562	0.343	0.762	0.794	0.761	0.471	0.701	0.682
MA-Net R50	C	10^{-4}	0.694	0.566	0.352	0.756	0.803	0.752	0.472	0.700	0.678
U-Net++ Inc-v4	C	10^{-4}	0.700	0.587	0.273	0.763	0.814	0.768	0.471	0.693	0.679
MA-Net Inc-v4	C	10^{-4}	0.699	0.567	0.271	0.768	0.816	0.760	0.469	0.695	0.677
DeepLabV3+ R50	C	10^{-4}	0.683	0.584	0.342	0.733	0.799	0.757	0.467	0.670	0.675
LinkNet R50	C	10^{-3}	0.702	0.480	0.331	0.768	0.814	0.748	0.467	0.700	0.672
M2F R50	T	10^{-4}	0.691	0.560	0.292	0.747	0.801	0.753	0.469	0.691	0.670
MobileSAM ViT-T	T	10^{-4}	0.666	0.564	0.277	0.731	0.800	0.754	0.467	0.692	0.684
UNet-GNRes	C	10^{-3}	0.703	0.567	0.169	0.771	0.811	0.766	0.471	0.698	0.669
UNet-GN	C	10^{-4}	0.702	0.555	0.162	0.767	0.803	0.768	0.460	0.695	0.690
M2F Swin-S	T	10^{-4}	0.699	0.552	0.178	0.760	0.792	0.763	0.464	0.687	0.672
M2F Swin-T	T	10^{-4}	0.701	0.553	0.091	0.761	0.804	0.764	0.468	0.684	0.700
SegRoot W8xD5	C	10^{-3}	0.685	0.553	0.086	0.735	0.803	0.730	0.455	0.685	0.664
SAM2 Hiera-B+	T	10^{-4}	0.660	0.122	0.196	0.716	0.581	0.652	0.380	0.592	0.617
SAM2 Hiera-S	T	10^{-3}	0.118	0.200	0.182	0.532	0.715	0.598	0.374	0.519	0.576

Additional file 5: Mean test Dice across datasets

Mean test Dice for each model across all nine datasets, using the best configuration selected by mean validation Dice. Models ordered by mean test Dice (best at left). Markers: × = Transformer, + = ConvNet.

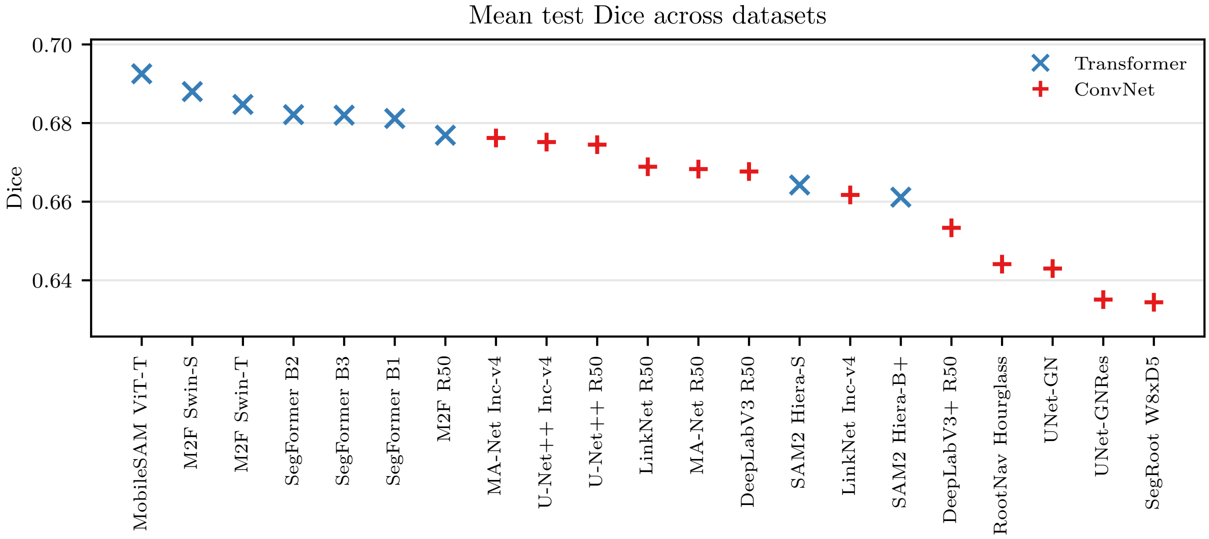

Additional file 6: Per-dataset test Dice breakdown

Test Dice for each model on each of the nine datasets. Same model ordering and configuration selection as Additional file 5. Markers: × = Transformer, + = ConvNet.

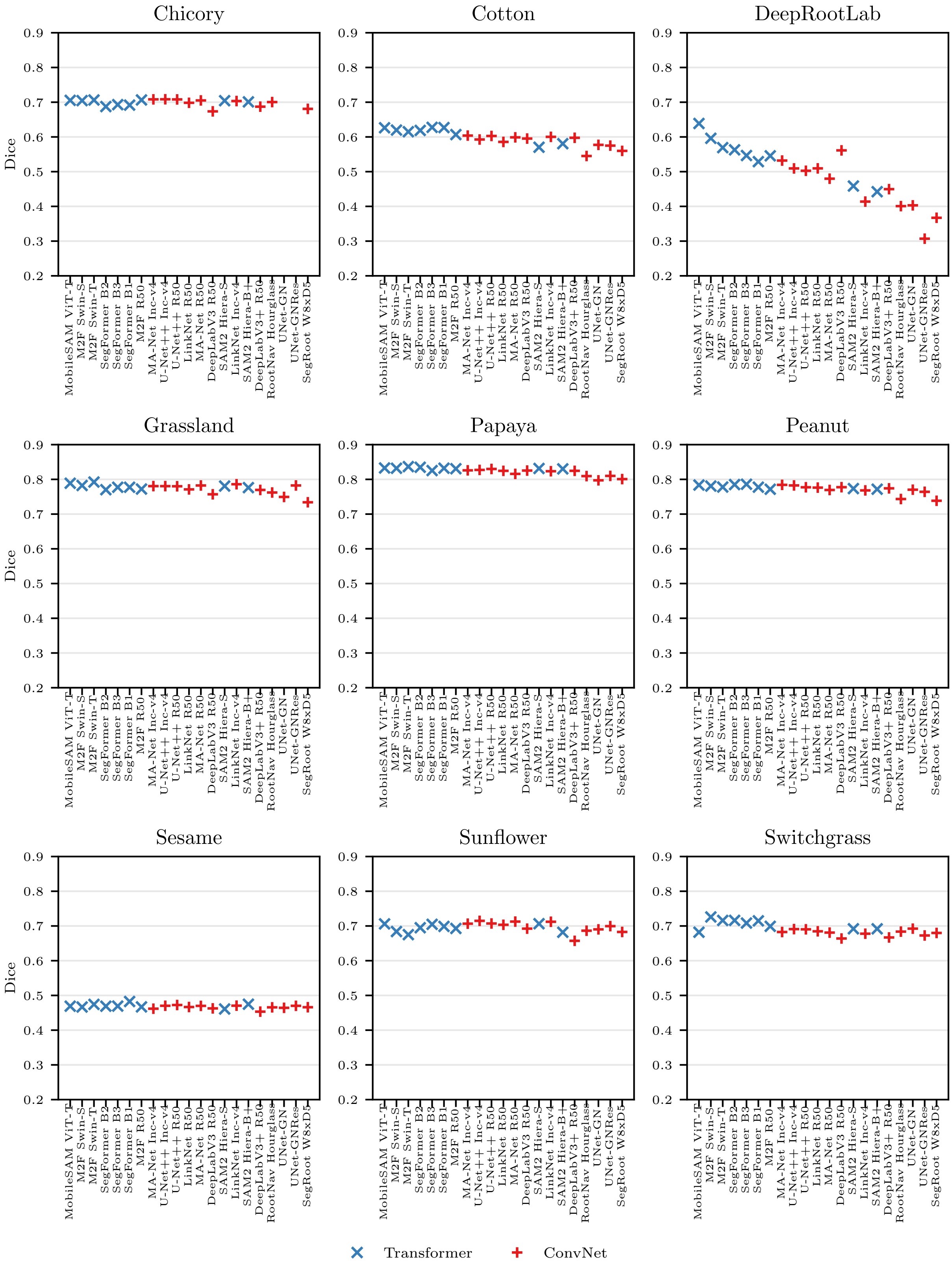
Chicory
Cotton
DeepRootLab
Grassland
Papaya
Peanut
Sesame
Sunflower
Switchgrass
Dice
0.2
0.3
0.4
0.5
0.6
0.7
0.8
0.9
MobileSAM ViT-T
M2F Swin-S
M2F Swin-T
SegFormer B2
SegFormer B3
SegFormer B1
M2F R50
MA-Net Inc-v4
U-Net++ Inc-v4
U-Net++ R50
LinkNet R50
MA-Net R50
DeepLabV3 R50
SAM2 Hiera-S
LinkNet Inc-v4
SAM2 Hiera-B+
DeepLabV3+ R50
RootNav Hourglass
UNet-GN
UNet-GNRes
SegRoot W8xD5
Transformer
ConvNet

Additional file 7: RhizoVision Explorer Configuration

Root phenotyping measurements were extracted using a fork of RhizoVision Explorer (RVE) v2.0 with patches for headless operation, memory safety, and command-line argument parsing. The fork and its modified dependency library are available at `https://github.com/sotlampr/RhizoVisionExplorer` (commit `a08860c`) and `https://github.com/sotlampr/cvutil` (commit `160d38c`).
RVE was invoked as: `rv -i -r -na -dranges 1,2,3,...,201`, where `-i` inverts the image (white roots on black background), `-r` enables recursive directory processing, `-na` prevents appending to existing output files, and `-dranges` specifies diameter bin boundaries from 1 to 201 pixels in 1-pixel increments.

Additional file 8: Patch size ablation

Test Dice for MobileSAM ViT-T (top-ranked Transformer) and MA-Net Inc-v4 (top-ranked ConvNet) at their original and swapped patch sizes. MobileSAM normally uses 1024 pixels, MA-Net normally uses 576 pixels. Both models trained with LR = 10^{-4}, pre-trained weights, averaged over two random seeds.

Dataset	MobileSAM ViT-T		MA-Net Inc-v4	
	1024 (orig.)	576 (swap.)	576 (orig.)	1024 (swap.)
Chicory	0.705	0.700	0.708	0.710
Cotton	0.626	0.620	0.604	0.612
DeepRootLab	0.639	0.600	0.532	0.502
Grassland	0.789	0.779	0.781	0.778
Papaya	0.833	0.833	0.826	0.828
Peanut	0.784	0.782	0.784	0.776
Sesame	0.469	0.464	0.462	0.474
Sunflower	0.706	0.703	0.707	0.700
Switchgrass	0.682	0.675	0.683	0.733
Mean	0.693	0.684	0.676	0.679
