# Supplementary material for: A systematic comparison of transformers and ConvNets for root segmentation across nine datasets
Source: Plant Methods. 2026 Apr 20;22:52. doi: 10.1186/s13007-026-01533-6 (PMC13227879; doi:10.1186/s13007-026-01533-6)

Additional File 9: Training Curves

A Systematic Comparison of Transformers and ConvNets for Root Segmentation Across Nine Datasets

Abraham George Smith, Sotiris Lamprinidis, Anand Seethepalli,
Larry M. York, Eusun Han, Patrick Möhl, Kyriaki Boulata,
Kristian Thorup-Kristensen, Jens Petersen

Description

Validation Dice curves during training for all 21 architectures on each of the nine datasets. Each page shows one dataset. Models are ordered by test Dice ranking (Table 3 in the main text). For each model, the best configuration (learning rate and pre-training) was selected by mean validation Dice across datasets, as described in Section 5.5 of the main text. Two curves per panel correspond to two training runs with different random seeds.

Blue = Transformer, red = ConvNet.

Chicory

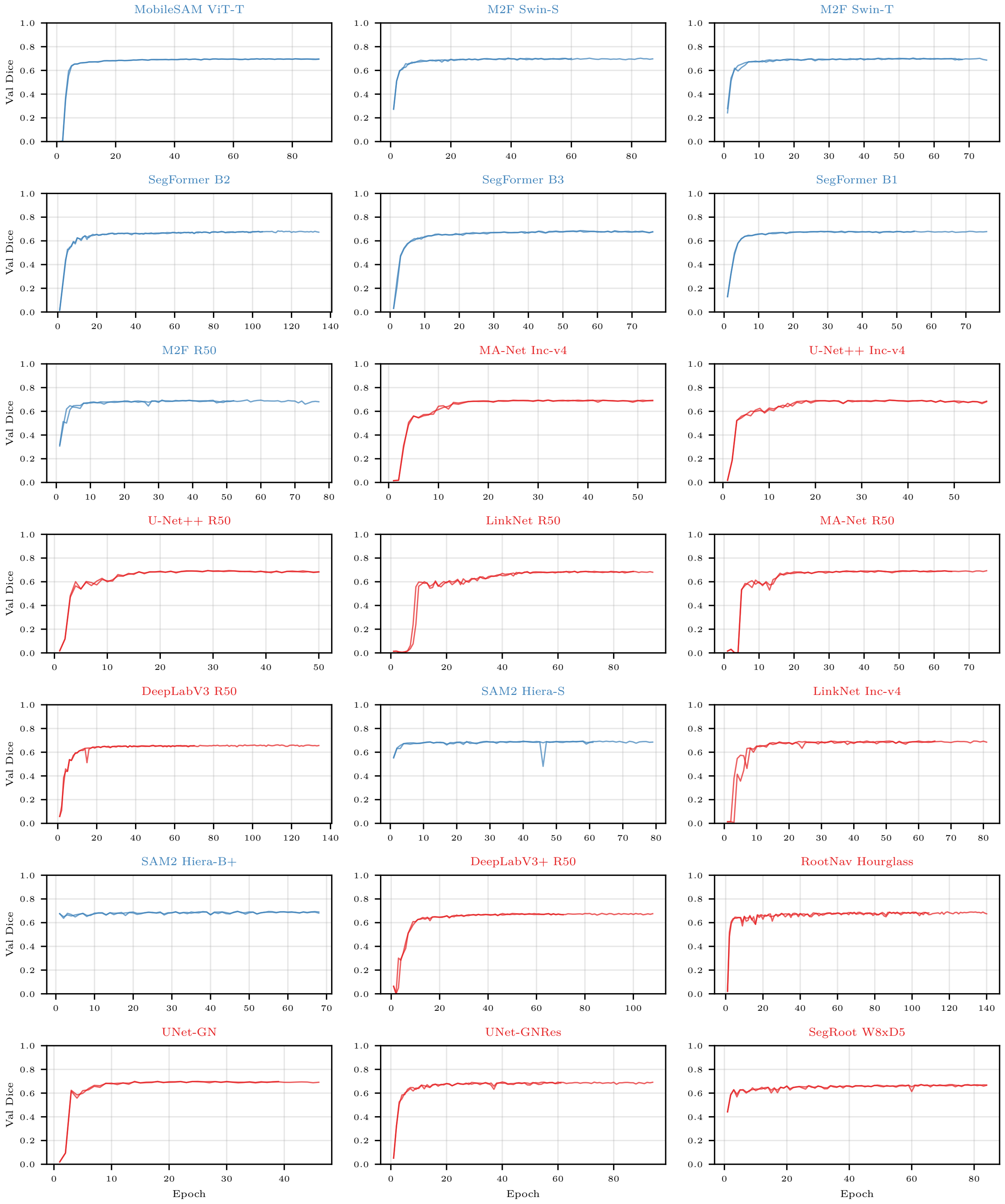

MobileSAM ViT-T
M2F Swin-S
M2F Swin-T
SegFormer B2
SegFormer B3
SegFormer B1
M2F R50
MA-Net Inc-v4
U-Net++ Inc-v4
U-Net++ R50
LinkNet R50
MA-Net R50
DeepLabV3 R50
SAM2 Hiera-S
LinkNet Inc-v4
SAM2 Hiera-B+
DeepLabV3+ R50
RootNav Hourglass
UNet-GN
UNet-GNRes
SegRoot W8xD5
Val Dice
Epoch

Cotton

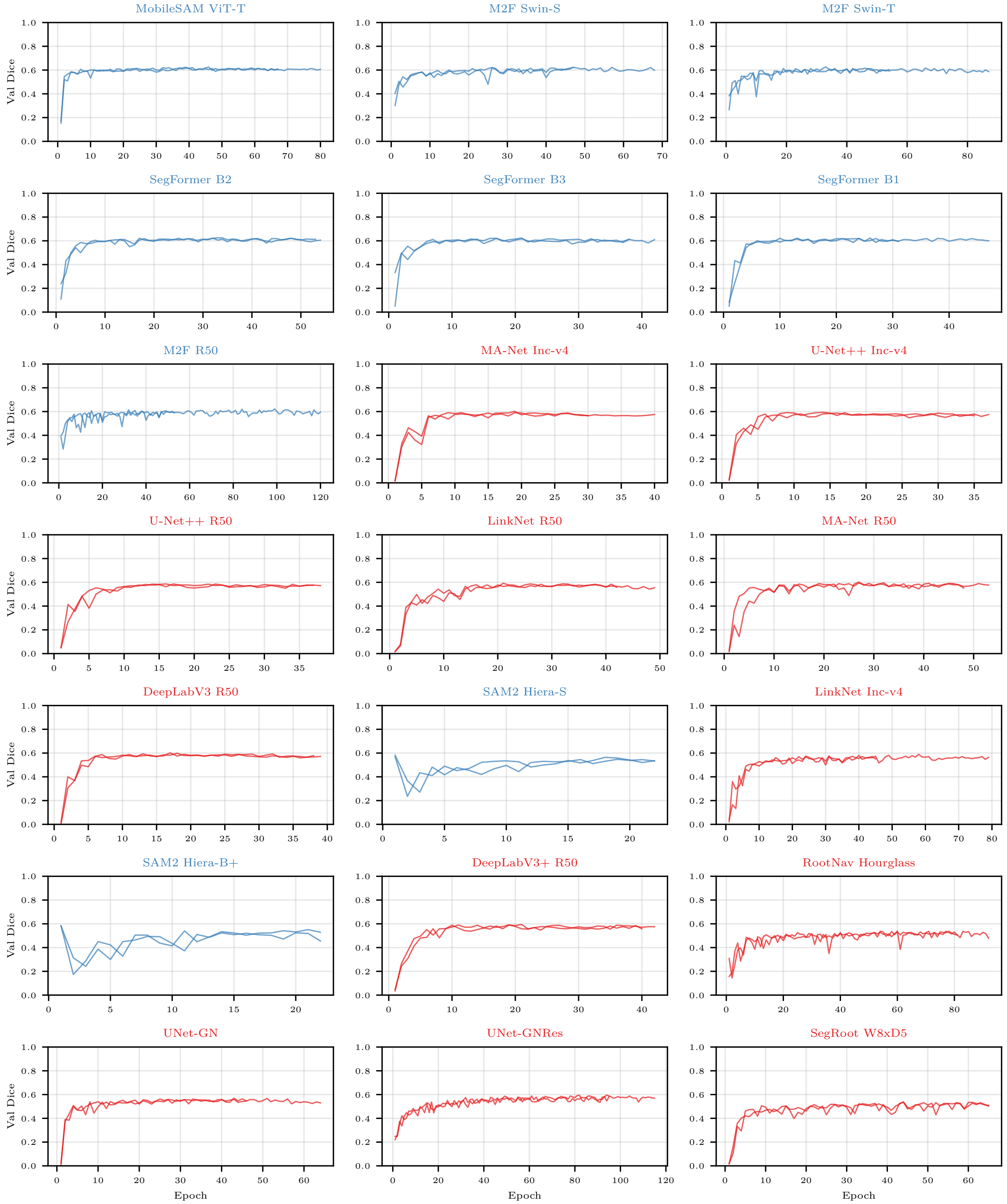
MobileSAM ViT-T
M2F Swin-S
M2F Swin-T
SegFormer B2
SegFormer B3
SegFormer B1
M2F R50
MA-Net Inc-v4
U-Net++ Inc-v4
U-Net++ R50
LinkNet R50
MA-Net R50
DeepLabV3 R50
SAM2 Hiera-S
LinkNet Inc-v4
SAM2 Hiera-B+
DeepLabV3+ R50
RootNav Hourglass
UNet-GN
UNet-GNRes
SegRoot W8xD5
Val Dice
Epoch

DeepRootLab

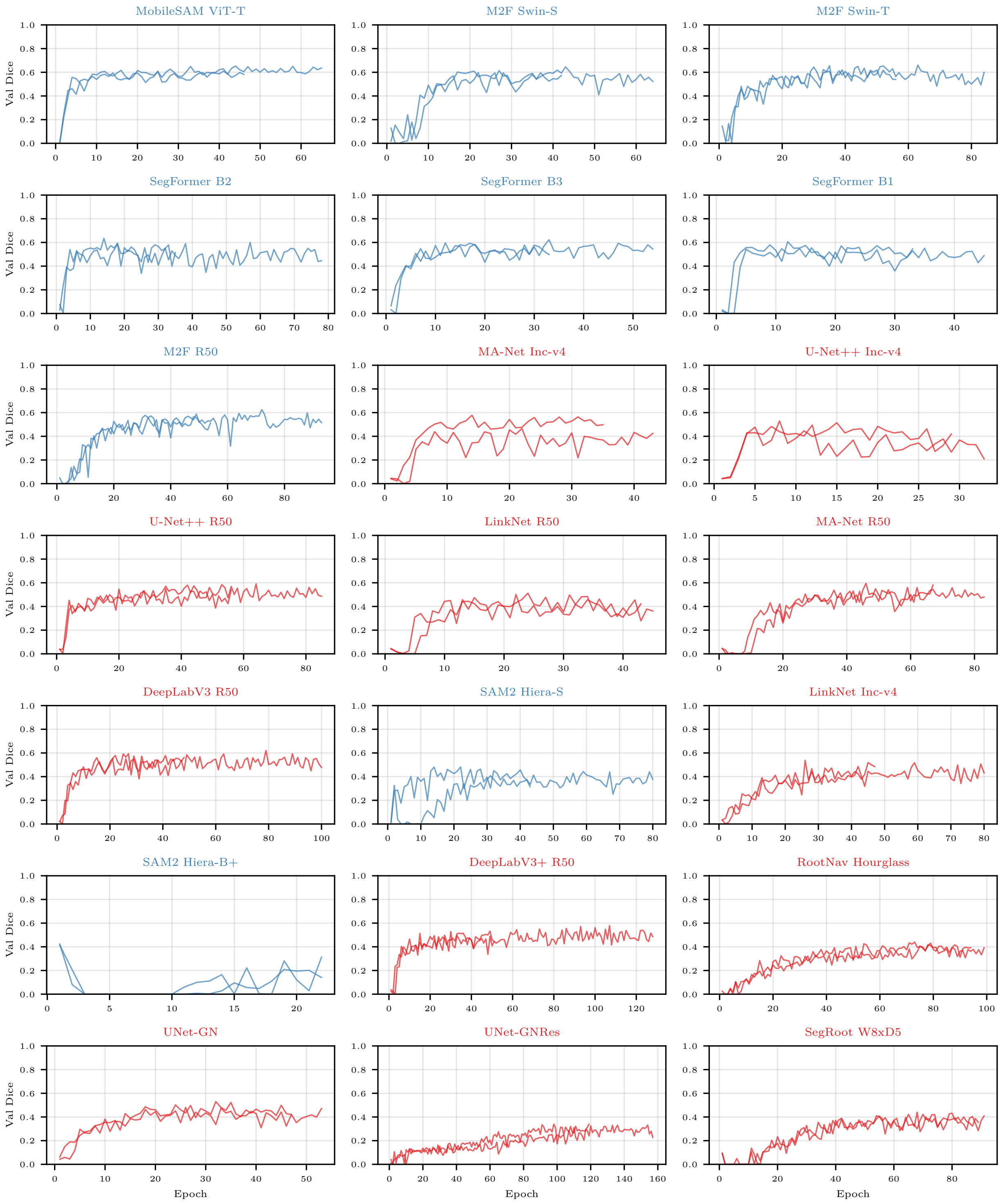

Grassland

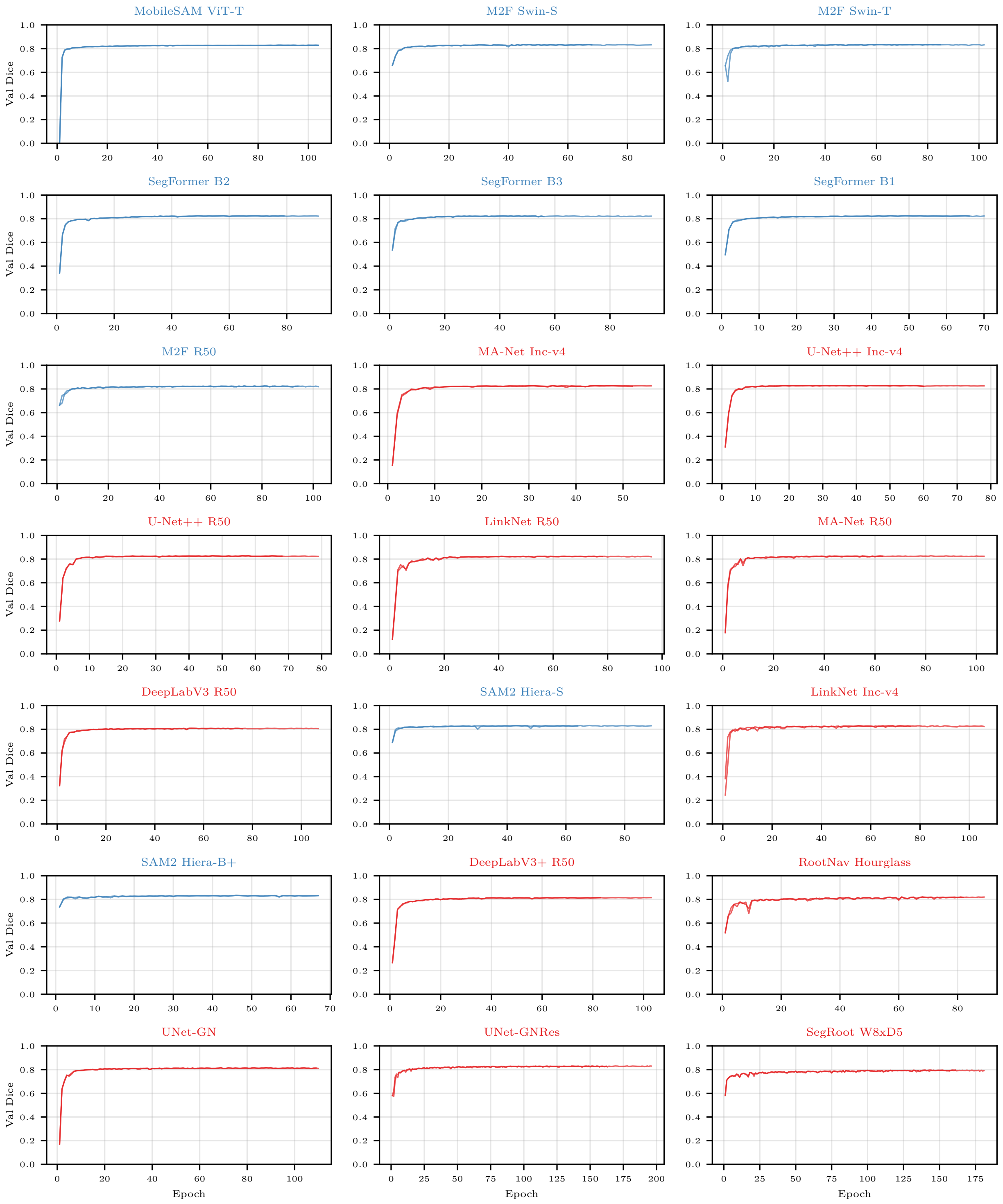

MobileSAM ViT-T
M2F Swin-S
M2F Swin-T
SegFormer B2
SegFormer B3
SegFormer B1
M2F R50
MA-Net Inc-v4
U-Net++ Inc-v4
U-Net++ R50
LinkNet R50
MA-Net R50
DeepLabV3 R50
SAM2 Hiera-S
LinkNet Inc-v4
SAM2 Hiera-B+
DeepLabV3+ R50
RootNav Hourglass
UNet-GN
UNet-GNRes
SegRoot W8xD5
Val Dice
Epoch

Papaya

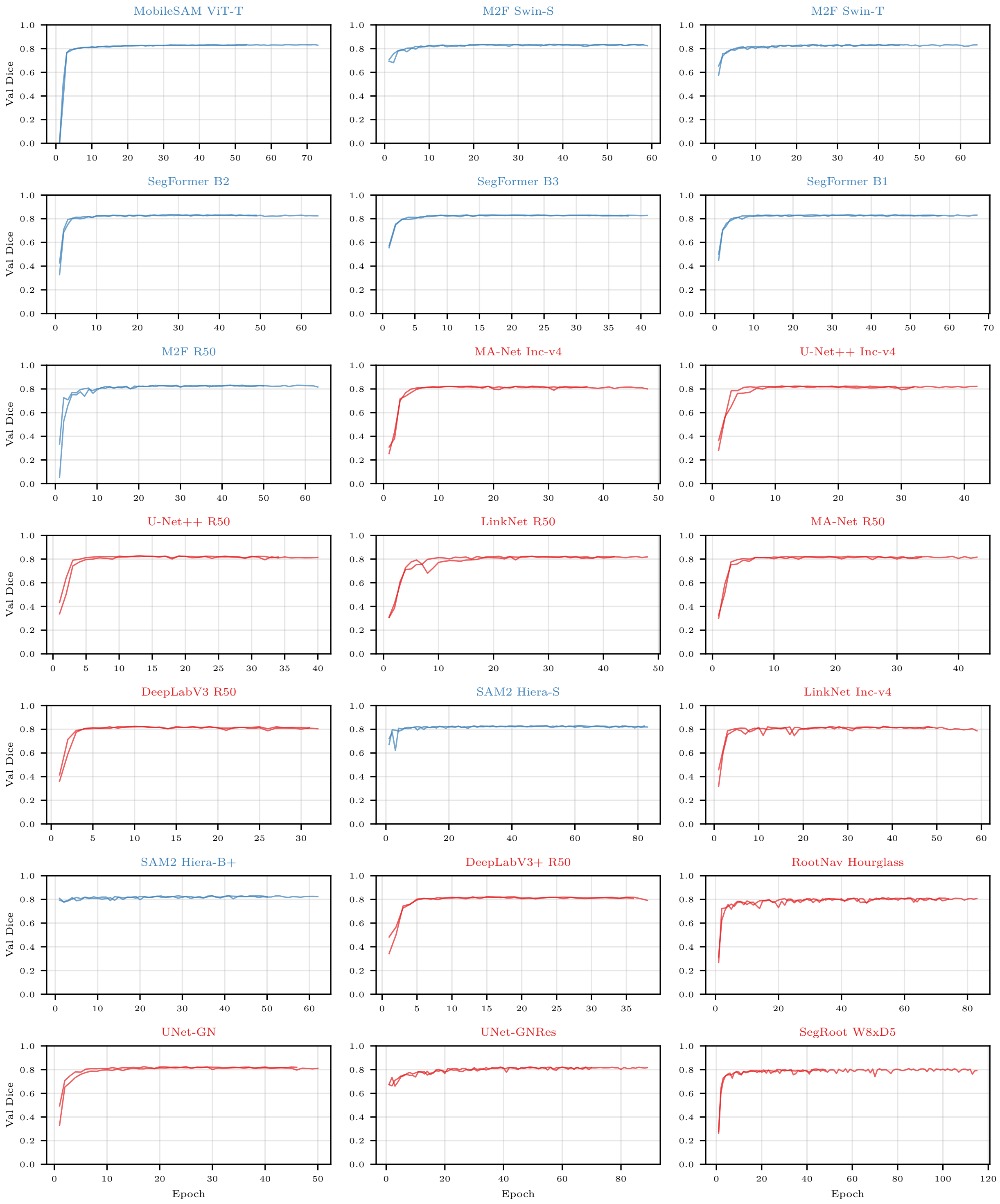

MobileSAM ViT-T
M2F Swin-S
M2F Swin-T
SegFormer B2
SegFormer B3
SegFormer B1
M2F R50
MA-Net Inc-v4
U-Net++ Inc-v4
U-Net++ R50
LinkNet R50
MA-Net R50
DeepLabV3 R50
SAM2 Hiera-S
LinkNet Inc-v4
SAM2 Hiera-B+
DeepLabV3+ R50
RootNav Hourglass
UNet-GN
UNet-GNRes
SegRoot W8xD5
Val Dice
Epoch

Peanut

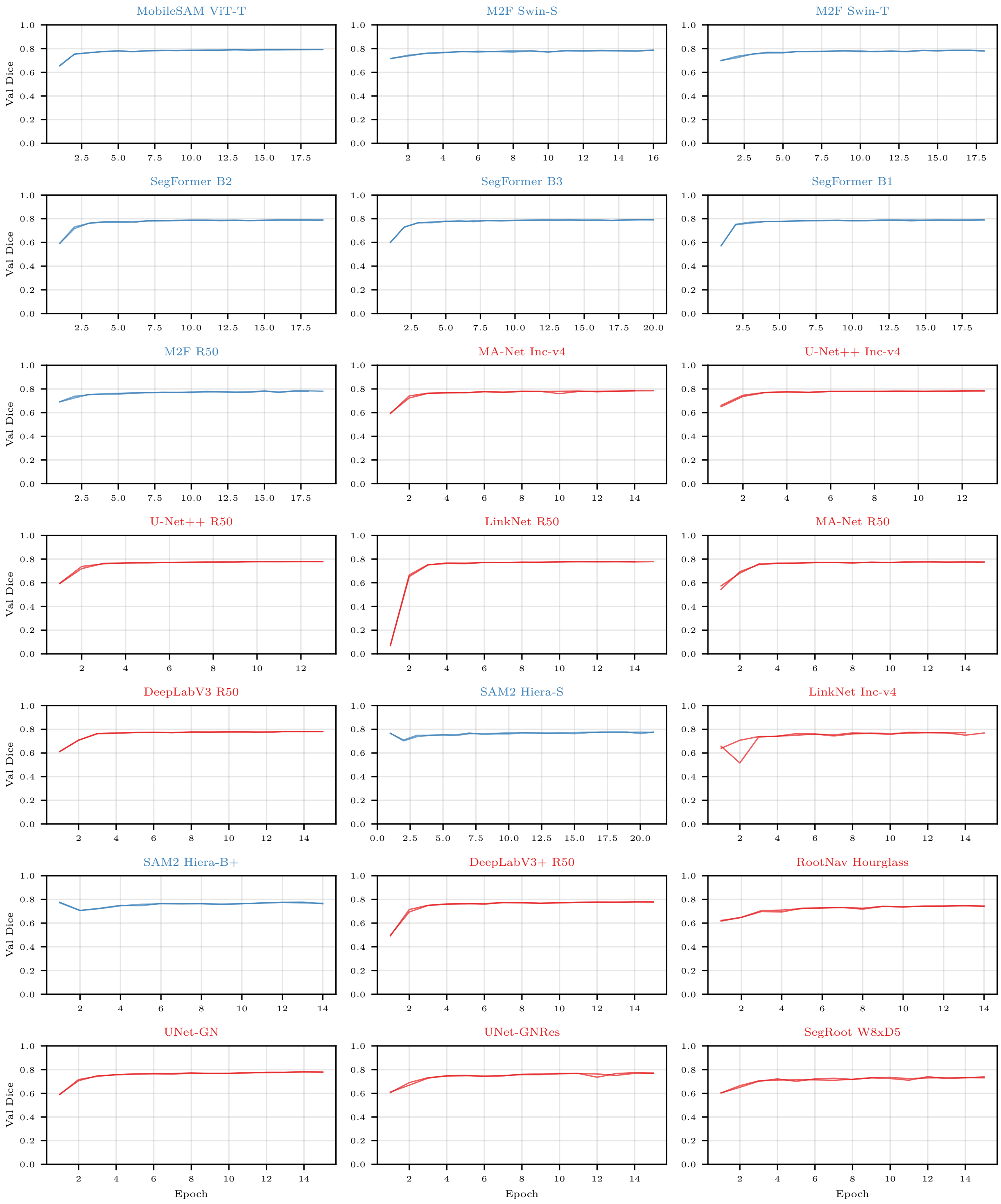

MobileSAM ViT-T
M2F Swin-S
M2F Swin-T
SegFormer B2
SegFormer B3
SegFormer B1
M2F R50
MA-Net Inc-v4
U-Net++ Inc-v4
U-Net++ R50
LinkNet R50
MA-Net R50
DeepLabV3 R50
SAM2 Hiera-S
LinkNet Inc-v4
SAM2 Hiera-B+
DeepLabV3+ R50
RootNav Hourglass
UNet-GN
UNet-GNRes
SegRoot W8xD5
Val Dice
Epoch

Sesame

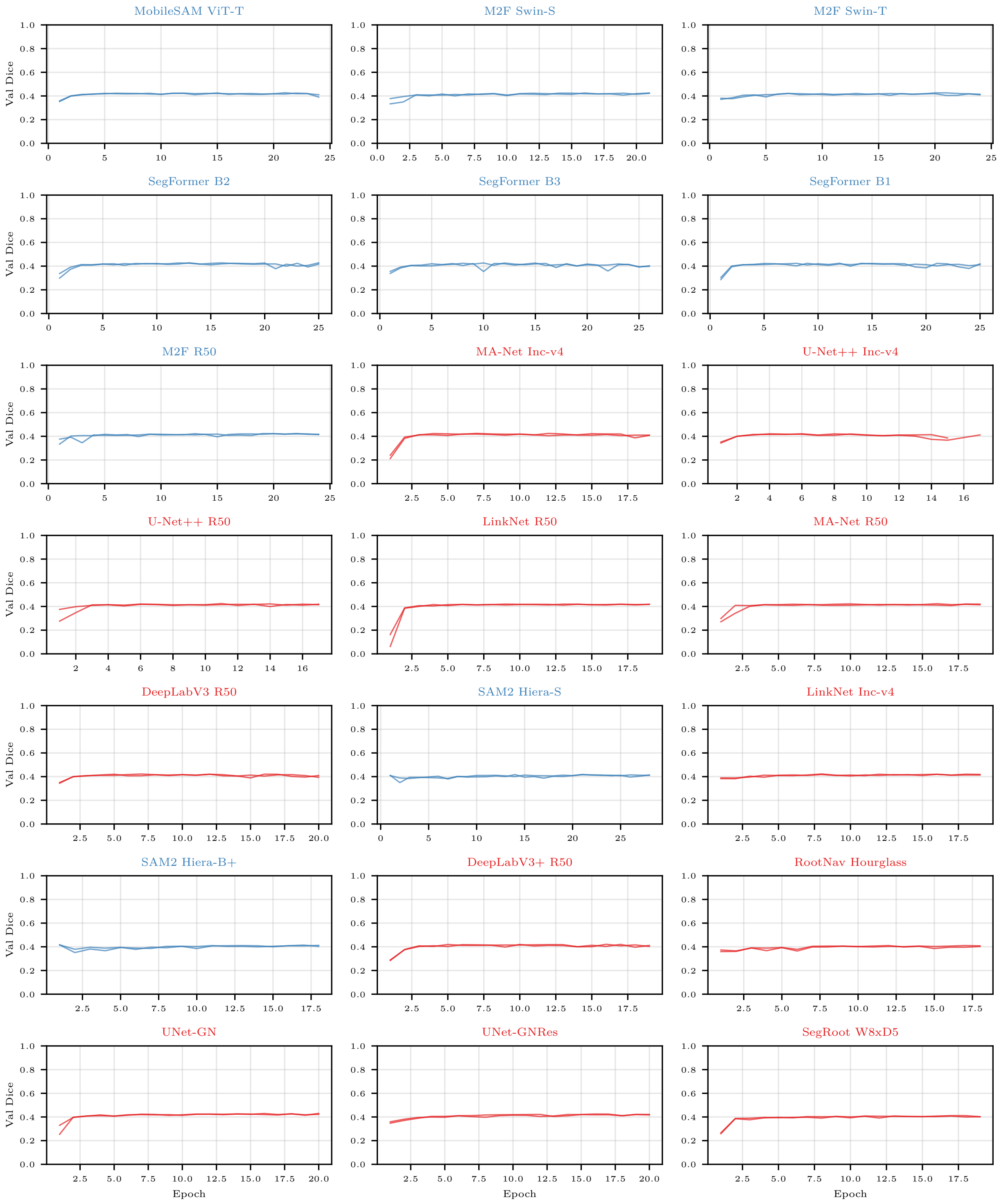
MobileSAM ViT-T
M2F Swin-S
M2F Swin-T
SegFormer B2
SegFormer B3
SegFormer B1
M2F R50
MA-Net Inc-v4
U-Net++ Inc-v4
U-Net++ R50
LinkNet R50
MA-Net R50
DeepLabV3 R50
SAM2 Hiera-S
LinkNet Inc-v4
SAM2 Hiera-B+
DeepLabV3+ R50
RootNav Hourglass
UNet-GN
UNet-GNRes
SegRoot W8xD5
Val Dice
Epoch

Sunflower

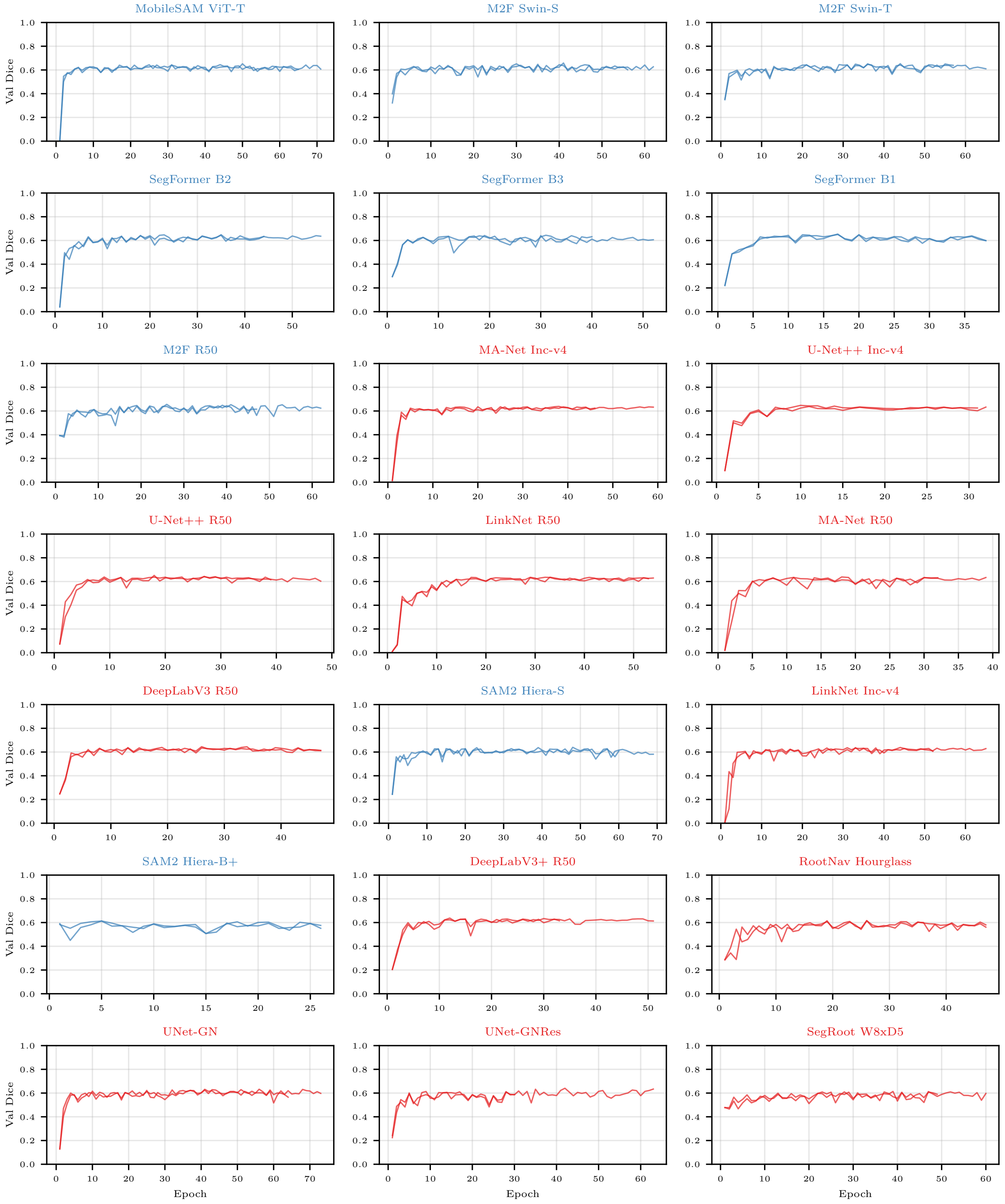

MobileSAM ViT-T
M2F Swin-S
M2F Swin-T
SegFormer B2
SegFormer B3
SegFormer B1
M2F R50
MA-Net Inc-v4
U-Net++ Inc-v4
U-Net++ R50
LinkNet R50
MA-Net R50
DeepLabV3 R50
SAM2 Hiera-S
LinkNet Inc-v4
SAM2 Hiera-B+
DeepLabV3+ R50
RootNav Hourglass
UNet-GN
UNet-GNRes
SegRoot W8xD5
Val Dice
Epoch

Switchgrass

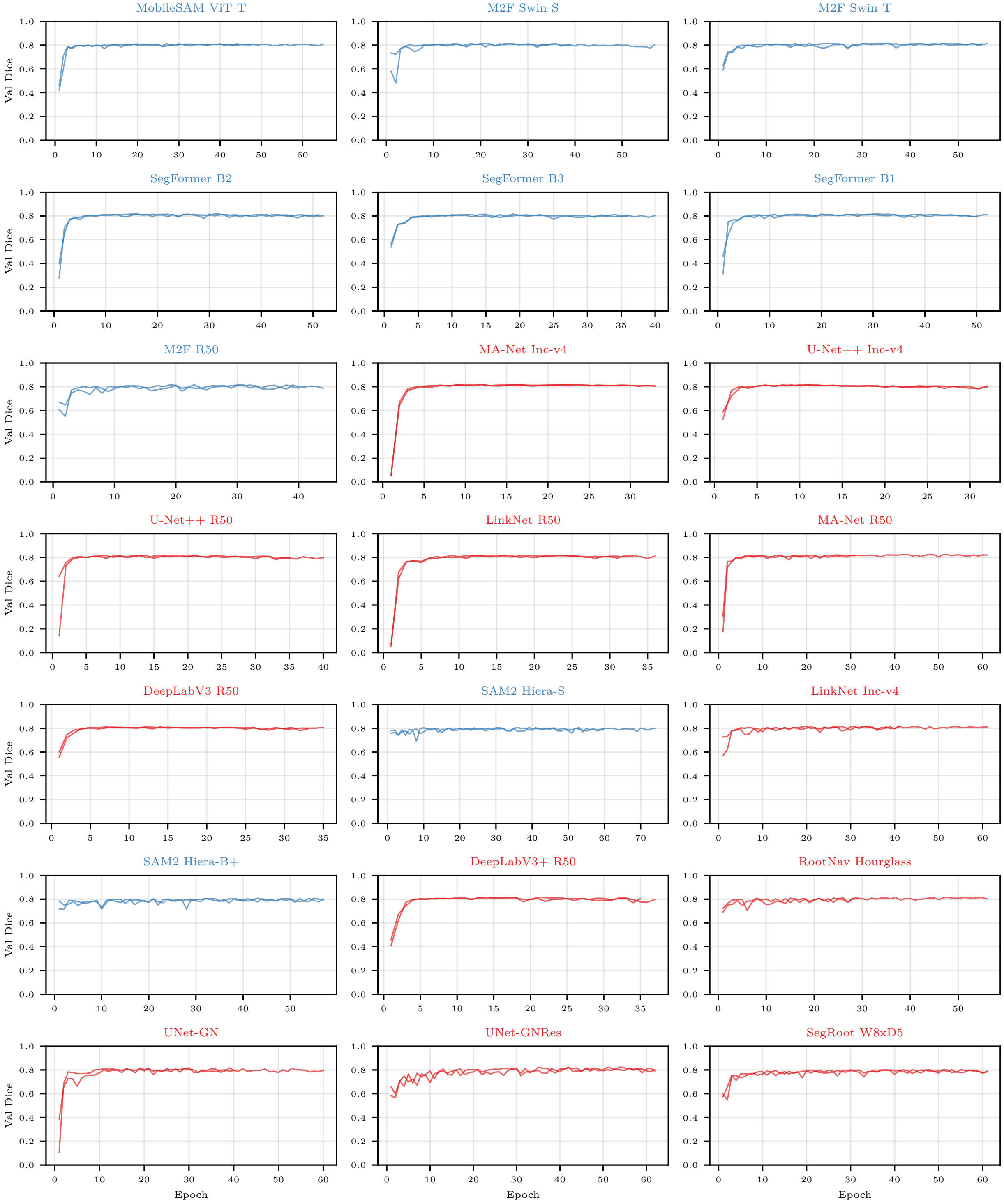

Supplement: Supplementary file 2 — Supplementary Material 2. [file 13007_2026_1533_MOESM2_ESM.pdf]
